# Management of Acquired Hypothalamic Dysfunction and the Hypothalamic Syndrome; It Is More Than Obesity

**DOI:** 10.1210/endrev/bnaf025

**Published:** 2025-08-01

**Authors:** Hanneke M van Santen, Hermann L Müller

**Affiliations:** Department of Pediatric Endocrinology, Wilhelmina Children's Hospital, UMC Utrecht, Utrecht 3508XB, the Netherlands; Princess Máxima Center for Pediatric Oncology, Heidelberglaan 25, Utrecht 3584CS, the Netherlands; Department of Pediatrics and Pediatric Hematology/Oncology, University Children's Hospital, Klinikum Oldenburg AöR, Oldenburg 26133, Germany

**Keywords:** hypothalamic syndrome, hypothalamic obesity, hypothalamus, personalized medicine

## Abstract

The hypothalamus is the key regulator of human energy balance. Hypothalamic dysfunction leads to (morbid) hypothalamic obesity, but may have many more consequences such as hypopituitarism, adipsia, disruption of the circadian rhythm, decreased energy expenditure, low core body temperature, and behavioral changes. Many patients with hypothalamic dysfunction experience chronic fatigue, increased daytime sleepiness, headaches, inactivity, and mood disorders, all of which may contribute to the development of obesity. Adipsic arginine vasopressin deficiency, severe hypothermia, uncontrollable hyperphagia, and severe mood disorders may require 24/7 management. Signs and symptoms may be severe or mild. Severe hypothalamic dysfunction is usually readily diagnosed, but less severe hypothalamic dysfunction is much harder to recognize because, among other things, of its multifaceted presentation. Through raising awareness and by better categorization of the different clinical signs and symptoms of hypothalamic dysfunction within different domains, the underlying cause for fatigue and obesity observed in patients with hypothalamic dysfunction may be better understood, which in turn, will open new perspectives on successful management options. In this review, the state of the art for diagnostics and management of acquired hypothalamic dysfunction is summarized and a new management algorithm is suggested. The lessons learned from pediatric patients with acquired hypothalamic dysfunction, including hypothalamic obesity management through the different clinical domains, may also prove to be useful for patients with congenital or genetic forms of hypothalamic dysfunction resulting in fatigue and obesity, as well as for children with presumed “common” obesity.

## Essential Points

- Hypothalamic syndrome is frequent after treatment for a suprasellar brain tumor in childhood and adolescence
- Hypothalamic syndrome can be devastating, requiring permanent and intense management due to uncontrollable hyperphagia, severe temperature spikes, adipsic arginine vasopressin (AVP) deficiency, and behavioral problems
- Hypothalamic dysfunction may present with different clinical manifestations and can be difficult to recognize
- Patients with hypothalamic dysfunction require multidisciplinary management in an expert setting
- A diagnostic score for hypothalamic dysfunction helps to recognize and individualize hypothalamic dysfunction
- Characterization of hypothalamic dysfunction in the different clinical domains opens new perspectives on improved management
- Improved treatment for suprasellar tumors must focus not only on survival but also on prevention of hypothalamic syndrome

## Hypothalamus and Its Functions

**Figure 1.**
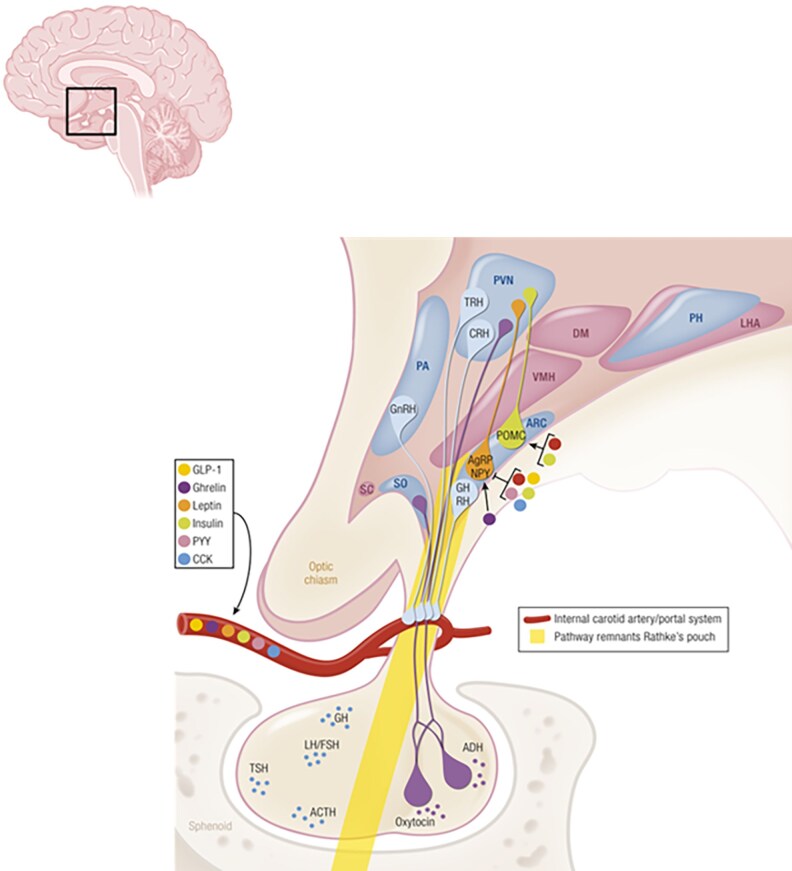
Schematic overview of the nuclei within the human hypothalamus, the satiety hormones, and its connection to the pituitary gland. The hypothalamic nuclei are interconnected through neural pathways; the connection between the arcuate nucleus (ARC) and paraventricular nucleus (PVN) is highlighted. Hypothalamic-releasing hormones (including thyrotropin-releasing hormone [TRH], corticotropin-releasing hormone [CRH], gonadotropin-releasing hormone [GnRH], and growth hormone–releasing hormone [GHRH]) are released into efferent blood vessels and stimulate the anterior pituitary gland to produce thyrotropin (TSH), adrenocorticotropin (ACTH), luteinizing hormone (LH)/follicle-stimulating hormone (FSH), and growth hormone (GH), respectively. Hunger and satiety hormones (such as ghrelin, leptin, insulin, and glucagon-like peptide-1 [GLP1]) stimulate the so-called orexigenic and anorexigenic responses, respectively, in hypothalamic neurons through afferent blood vessels. ADH, antidiuretic hormone; AgRP, agouti-related peptide; CCK, cholecystokinin; NPY, neuropeptide Y; POMC, pro-opiomelanocortin; PYY, polypeptide-Y. The connection between the ARC and PVN is emphasized. AgRP/NPY, agouti-related peptide neuropeptide Y; CRH, corticotropin-releasing hormone; DM, dorsomedial hypothalamic nucleus; LHA, lateral hypothalamic area; OC, optic chiasm; PA, preoptic area; PH, posterior hypothalamic nucleus; PYY, polypeptide-Y; SC, suprachiasmatic nucleus; SO, supraoptic nucleus; VMN, ventromedial nucleus. Reproduced from van Iersel et al, 2019 (3).

**Figure 2.**
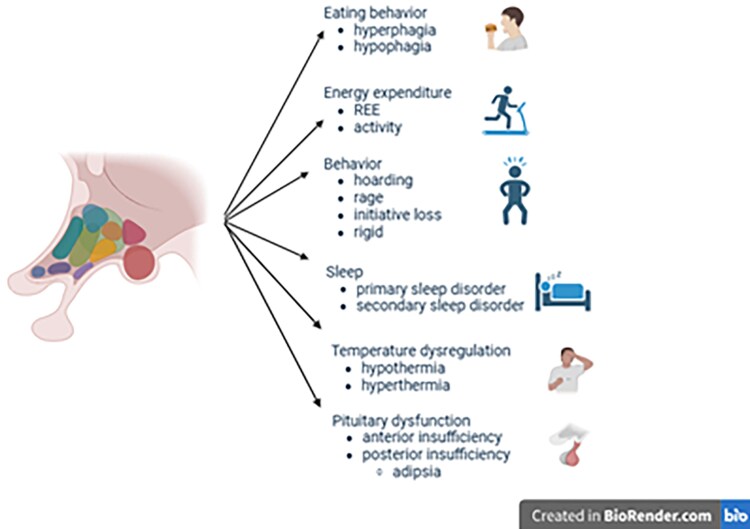
The 6 different domains in which individuals with hypothalamic dysfunction may experience signs or symptoms. Dysfunction in any of these domains may cause or contribute to the development of chronic fatigue, obesity, increased daytime sleepiness, inactivity, mood disorders, and the inability to keep up with peers.

The hypothalamus is a small, ancient neuroendocrine organ in the brain that contains several nuclei responsible for regulating the body’s balance (1, 2) (Fig. 1). The hypothalamus may be regarded as a “control center” with outputs to the pituitary gland (hormones), to the autonomic nervous system, and to other areas in the brain such as the frontal lobe, through its connective circuits (behavior). Hypothalamic dysfunction leads to (morbid) obesity or cachexia, but may have many more clinical consequences such as hypopituitarism, adipsia, disruption of the circadian rhythm, decreased energy expenditure, disturbed regulation of core body temperature, and behavioral changes (Fig. 2). Many patients with hypothalamic dysfunction experience chronic fatigue (4), increased daytime sleepiness (5), headaches (6), inactivity (7), and mood disorders (8), all of which may contribute to the development of obesity. Patients with hypothalamic dysfunction experience that their symptoms are not always adequately recognized or addressed (9), which may be due to the nonspecificity of its signs and symptoms as well as the rareness of hypothalamic dysfunction. Improvement of care is needed, to decrease the diagnostic delay of hypothalamic dysfunction as well as to improve the consequences of hypothalamic dysfunction such as (morbid) obesity and (chronic) fatigue. Adipsic arginine vasopressin (AVP) deficiency (10), severe hypothermia and/or hyperthermia (11), uncontrollable hyperphagia (12) and severe mood disorders (8) may require 24/7 management.

### Hypothalamus and Energy Balance

Energy balance is determined by the relationship between energy intake and energy expenditure, which is managed by various hypothalamic neural circuits that integrate both peripheral and central signals (3, 11). Energy intake is influenced by 3 key processes: initiating meals, stopping meals, and making food choices. Meal initiation is controlled by the hypothalamus through neuroendocrine hormones, which are categorized into orexigenic (hunger-stimulating: ghrelin, neuropeptide Y, orexin A and B) and anorexigenic (hunger-suppressing: leptin, insulin, adiponectin, brain-derived neurotrophic factor) signals. Meal termination is guided by the hypothalamus responding to hormones from the gut, while food choice is influenced by the mesolimbic reward system and decision-making circuits in areas like the prefrontal cortex and amygdala, which connect with the hypothalamus. The total energy expenditure is the sum of the resting metabolic rate (60%-75%), the activity-induced thermogenesis (∼20%), and the diet-induced thermogenesis (10%-15%). The sympathetic nervous system is involved in all these aspects of energy expenditure, with the hypothalamus playing a role in its regulation. If the hypothalamus is damaged, it can impair parasympathetic signaling, leading to hyperinsulinemia. This increased insulin secretion promotes calorie storage in fat cells, contributing to weight gain.

Disruptions in the hypothalamus can disturb the body's energy balance in different ways (13). These disruptions can affect the classical endocrine circuits (like the hypothalamic-pituitary axes), the neuroendocrine circuits, or the neural pathways that connect these systems or that are connected to other regions of the brain (14). These disruptions can be partial or complete and may vary depending on the patient's age, underlying conditions, and other health factors. The mechanisms behind the hypothalamus's role in appetite and weight regulation are complex, but research is gradually uncovering more details (15). An important pathway that regulates eating behavior is the melanocortin pathway. After feeding, leptin binds to the leptin receptor, which is expressed on pro-opiomelanocortin (POMC) neurons in the hypothalamus. After binding, POMC is expressed, which leads to the forming of α- and β-melanocyte–stimulating hormones (α- and β-MSH), which in turn activate the melanocortin-4 receptor to promote satiety and increase energy expenditure. In cases where this pathway is distorted, severe hunger feelings with increased calory intake (hyperphagia) will be present, leading to obesity (15). Measuring the neuroendocrine hormones associated with appetite regulation (like leptin, insulin, and ghrelin) has not yet led to a reliable test for diagnosing hypothalamic dysfunction. This may be explained by the fact that hormone concentrations in the blood, such as agouti-related peptide (AGRP) and neuropeptide Y (NPY), may not accurately reflect brain levels, can vary greatly between individuals, and are influenced by factors like circadian rhythms, nutritional status, and body composition (15).

As explained earlier, the efferent hypothalamic pathway influences the sympathetic nervous system, which contributes to energy expenditure. Indeed, a lower energy expenditure is measured in children with hypothalamic dysfunction. In a cohort of 67 children with hypothalamic dysfunction, 67.2% had a measured resting energy expenditure (mREE) less than 90% than predicted (pREE), and children with severe hypothalamic dysfunction had a significantly lower mean mREE/pREE quotient compared to children with no, mild, or moderate hypothalamic dysfunction (16). Also, the mean mREE/pREE quotient of children with posterior hypothalamic damage was significantly lower compared to children with no or anterior damage, which correlates to the fact that children with posterior hypothalamic damage have the most severe hypothalamic obesity (16). In addition, REE is also related to the percentage of muscle mass (17), due to the fact that muscle mass is more metabolically active when compared to fat mass. Physical exercise will thus increase the resting energy expenditure by two mechanisms; first directly by increasing muscle mass and secondly by increasing activity-induced thermogenesis and thereby REE. In patients with hypothalamic obesity and little physical exercise, the percentage of muscle mass when compared to free fat mass may be low (18).

### Hypothalamus and Pituitary Function

The hypothalamus controls the pituitary gland through its releasing hormones, including growth hormone (GH)-releasing hormone, gonadotropin-releasing hormone, thyrotropin-releasing hormone, and corticotropin-releasing hormone. Patients with hypothalamic dysfunction may experience issues such as GH deficiency, central hypothyroidism, central hypogonadism, and/or central hypocortisolism (19). Additionally, the hypothalamus produces AVP, which is stored in the posterior pituitary gland. Those with hypothalamic dysfunction may suffer from AVP deficiency (formerly known as central diabetes insipidus (20)) or the syndrome of inappropriate AVP secretion (SIADH). Since the hypothalamus also regulates thirst, severe hypothalamic dysfunction may lead to adipsic AVP deficiency or adipsic SIADH, conditions that severely affect serum sodium levels and quality of life (7). Such conditions require constant monitoring of fluid balance or body weight and regular blood tests to check sodium levels. In young children with suprasellar tumors, hypothalamic dysfunction may result in the overproduction of pituitary hormones, resulting in central precocious puberty or increased concentrations of insulin-like growth factor (IGF)-1 (21).

### Hypothalamus and Sleep

**Figure 3.**
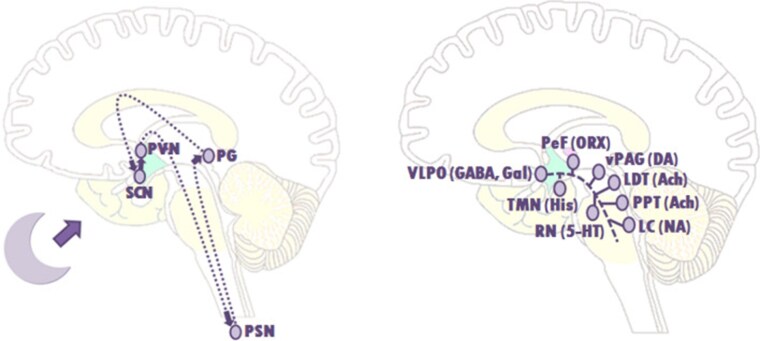
Schematic drawing of the circadian rhythm and arousal system involved in the flip-flop switch. Schematic drawing of the suprachiasmatic nuclei (SCN) controlling the circadian rhythm during sleep. Through activation of the SCN via the axons of retinal ganglion cells running in the optic nerves (forming a retinohypothalamic tract), the SCN sends a signal to the paraventricular nuclei (PVN) and preganglionic sympathetic neurons (PSN), which in their turn activate the pineal gland (PG) to produce melatonin. A schematic drawing to show the basic route of the ventrolateral preoptic nucleus (VLPO) to the main elements of the ascending arousal system, involved in the flip-flop switch. The arousal system consists of monoaminergic cell groups (MCGs) such as the tuberomammillary nucleus (TMN), the raphe nuclei (RN), and the locus coeruleus (LC). VLPO neurons also stimulate the neurons in the lateral hypothalamus, such as the perifornical (PeF) and orexin (ORX) neurons, and the cholinergic (ACh) cell groups; the pedunculopontine (PPT) and laterodorsal tegmental nuclei (LDT). During a wakeful state, the MCG inhibit the VLPO neurons, thereby activating the arousal system through the MCG, and that of the ORX neurons and the PPT and LDT. During sleep, the firing of the VLPO neurons suppress the MCG. This also allows it to inhibit the orexin neurons, further preventing monoaminergic activation that might interrupt sleep. The direct inhibition between both groups, the VLPO and the MCGs, forms the flip-flop switch. 5-HT, serotonin; GABA, γ-aminobutyric acid; gal, galanin; NA, noradrenaline; His, histamine (25). Reproduced from van Schaik et al 2020.

The suprachiasmatic nuclei are central to regulating the circadian rhythm, which controls sleep and wakefulness. Melatonin, produced by the pineal gland during the night, plays a significant role in regulating this rhythm. In children with hypothalamic dysfunction, the circadian rhythm may be disrupted due to altered melatonin secretion or a diminished response to it (22-24). Besides the suprachiasmatic nuclei, sleep is also regulated by the ventrolateral preoptic nucleus (VLPO), the lateral hypothalamus area (which promotes sleep), and monoaminergic cell groups (MCGs) that are part of the arousal system. These sleep-promoting and arousal systems can interact through a “flip-flop” switch, facilitating a quick transition between wakefulness and sleep (23) (Fig. 3). Damage to the VLPO can lead to insomnia, while damage to the lateral hypothalamus area can cause both disrupted sleep and excessive daytime sleepiness (26-29). Poor sleep quality may lead to headache, chronic fatigue, increased daytime sleepiness, and obesity. Children with hypothalamic dysfunction may have “primary” hypothalamic sleep dysfunction, such as hypersomnia, narcolepsy (29) and disturbed sleep-wake cycles (25). It is important to be aware of the fact that in children with hypothalamic damage following a brain tumor, with severe obesity or with visual impairment, “secondary” sleep disturbances may be present as well as psychosocial factors such as obstructive sleep apnea, badly regulated AVP deficiency (nocturia), or other medical factors (25).

### Hypothalamus and Temperature Regulation

The hypothalamus controls our core body temperature through various pathways, including the arcuate nucleus (which increases heat production), the preoptic area–dorsomedial nucleus of the hypothalamus circuit (which receives sensory input from temperature-sensitive neurons), and the ventromedial hypothalamus (which is involved in cold-induced thermogenesis) (11). In patients with hypothalamic dysfunction, temperature regulation may be disrupted, which may lead to severe hypothermia (<36 °C) or spikes of hyperthermia (>37.5 °C) (19, 30, 31). Also, core body temperature may be normal, but patients may have feelings of distorted temperature dysregulation with cold hands and feet but warm cheeks and upper body.

### Hypothalamus and Behavior

**Figure 4.**
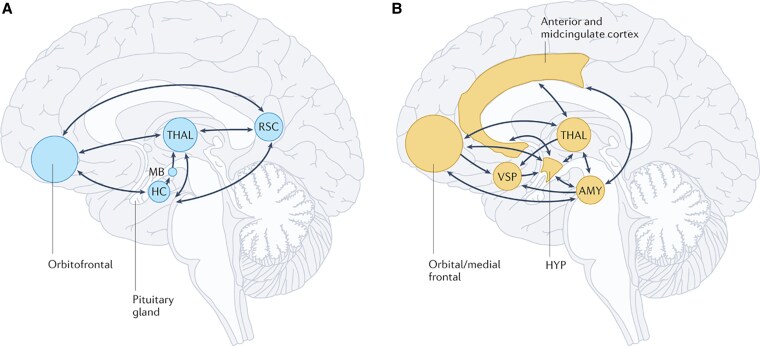
The hypothalamus (HYP) is an integral part of two different networks of the limbic system: A, a hippocampus (HC)-centered network essential for episodic memory and B, an amygdala (AMY)-centered network relevant for social–emotional functioning. Damage to brain regions within these networks or to their connecting fibers contributes to the neurobehavioral and psychiatric abnormalities in hypothalamic syndrome (HS). A, Episodic memory deficits in HS usually result from lesions to the mammillary bodies (MB) in the posterior part of the hypothalamus or their connecting fibers: fornical fibers projecting from the hippocampus to the MB, or fibers of the mammillothalamic tract projecting from the MB to the anterior thalamic nucleus. B, Deficits in social-emotional functioning in HS may result from lesions to hypothalamic nuclei anterior to the MB and, for example, from tumor-related or treatment-related damage to other regions of the AMY-centered network. RSC, retrosplenial cortex; THAL, thalamus; VSP, ventral striatopallidum (19, 30). Reproduced from Müller et al 2022.

The hypothalamus is connected to important brain areas within the limbic system that regulate behavior and the mind (Fig. 4). Dysfunction of the hypothalamus may thus also lead to mild or severe neurobehavioral and psychiatric abnormalities (19). Anxiety problems are commonly observed that may be caused by damage to the Papez circuit (32). Memory problems may be the consequence (8, 33). Damage to the nucleus accumbens may lead to addiction or obsession, hoarding behavior, or collecting items in excessive amounts (8). In combination with a disorder of the satiety pathways leading to hyperphagia, this may lead to uncontrollable eating behavior and morbid obesity.

## Diagnostic Criteria for Hypothalamic Dysfunction and Hypothalamic Syndrome

### Definition of Hypothalamic Syndrome

When the hypothalamus malfunctions, one speaks about hypothalamic dysfunction. Dysfunction of the hypothalamus may result in signs and symptoms in any of the aforementioned domains of hypothalamic function. Hypothalamic syndrome may be considered as “the worst form of hypothalamic dysfunction” and as an umbrella term that is characterized by intractable weight gain associated with morbid obesity, multiple endocrine abnormalities, and memory impairment, attention deficit, and reduced impulse control as well as increased risk of cardiovascular and metabolic disorders (19).

### Diagnostic Criteria for Hypothalamic Dysfunction and Hypothalamic Syndrome

Severe hypothalamic dysfunction, or hypothalamic syndrome, is readily diagnosed. However, because the clinical signs or symptoms of hypothalamic dysfunction can be nonspecific or less severe, it may be missed as an underlying cause for obesity or fatigue. For this reason, a clinical diagnostic score has been proposed for the diagnosis of hypothalamic dysfunction, and its most severe form, hypothalamic syndrome (Table 1) (30). To use diagnostic criteria for signs and symptoms of hypothalamic dysfunction may not only aid in early recognition, but is also useful for reporting and understanding the etiology as well as the management of hypothalamic syndrome. The diagnostic criteria have been subdivided into different categories: hyperphagia/hypophagia, body mass index (BMI), behavioral problems, sleep disorders, temperature regulation disorders, pituitary dysfunction, radiological assessment of hypothalamic lesions/integrity, and presence/suspicion of a hypothalamic genetic syndrome. In a first retrospective analysis including 120 patients with acquired hypothalamic dysfunction, 52% were scored as having hypothalamic syndrome (30). Of these patients, 77% were diagnosed with pituitary dysfunction, 32% with hyperphagia, 40% with sleep disorders, and 14% with temperature dysregulation. For several criteria, however, clinical data were missing in more than 50% of cases, emphasizing the importance of assessing the presence of hypothalamic dysfunction using these criteria in future prospective cohorts. To improve insight and knowledge on prevalence, risk factors for, and consequences of the different aspects of hypothalamic syndrome, it is strongly recommended to use standardized prospective data collection protocols. Uniform application of the diagnostic criteria of hypothalamic syndrome in future studies may not only facilitate broader validation of these criteria but also improve comparability of cohorts across centers.

**Table 1.** Adapted table for scoring the presence of signs and symptoms of hypothalamic dysfunction; clinical hypothalamic score (30)

Clinical criteria		Score	Total score*^c^*
Domain 1	Hyperphagia*^a^*	0 = No	1 = Minor criterion
		1 = Mild	2 = Major criterion
		2 = Mild after specific intervention for hyperphagia OR severe	
	Hypophagia/failure to thrive (diencephalic syndrome)	0 = No	1 = Minor criterion
		1 = Mild	2 = Major criterion
		2 = Severe	
Domain 2	BMI	0 = Normal weight or overweight	2 = Major criterion
		2 = Normal weight OR overweight after specific intervention for hypothalamic obesity OR obesity	
Domain 3	Behavioral problems*^b^*	0 = None	1 = Minor criterion
		1 = Mild	2 = Major criterion
		2 = Mild after specific intervention OR severe	
Domain 4	Sleep disorder	0 = no, Epworth Scale score 0-10	1 = Minor criterion
		1 = Mild	2 = Major criterion
		2 = Mild after specific intervention such as melatonin OR severe	
Domain 5	Temperature regulation disorder	0 = No	1 = Minor criterion
		1 = Mild	2 = Major criterion
		2 = Severe	
Domain 6	Pituitary dysfunction	0 = No pituitary dysfunction	1 = Minor criterion
		1 = Partial or complete pituitary dysfunction	2 = Major criterion
		2 = Pituitary dysfunction including AVP deficiency AND adipsia	

Legend to Table 1 (Adapted from van Santen et al 2023 (30)).

Abbreviations: AVP, arginine vasopressin; BMI, body mass index.

^*a*^Thirteen-item Dykens Hyperphagia Questionnaire.

^*b*^PWS Behavioral Questionnaire (PWSBQ) or neuropsychological investigation.

^*c*^Hypothalamic syndrome is considered present in case of 3 or more major criteria OR “at least” 4 minor criteria OR 2 major and “at least” 2 minor criteria.

## Causes and Prevalence of Acquired Hypothalamic Dysfunction in Childhood and Adolescence

Causes of hypothalamic dysfunction in childhood and adolescence can be congenital (eg, septo-optic dysplasia (SOD) syndrome (34)), genetic (eg, Prader-Will syndrome (PWS) (35)), acquired (eg, due to craniopharyngioma (19), low-grade glioma (LGG) (36), traumatic brain injury (TBI) (37) or unknown cause such as in rapid-onset obesity with hypothalamic dysregulation, hypoventilation, and autonomic dysregulation (ROHHAD) syndrome (38)). Prevalence of hypothalamic dysfunction in these cohorts ranges from 20% to 100% depending on its etiology, age of the cohort, follow-up time, and neurosurgical intervention that has been performed (19). In this review, the focus will be on the acquired forms of hypothalamic dysfunction.

### Adamantinomatous Craniopharyngioma

Childhood-onset craniopharyngioma (cCP) is one of the most described tumors to cause hypothalamic dysfunction (39). cCP is a rare tumor (40), and although it is considered to be benign or low-grade of origin (WHO I^0^), it may have devastating consequences for the patient. cCP presumably derives from ectodermal-derived epithelial cell remnants of the Rathke pouch. There are 2 distinct types: adamantinomatous (ACP) and papillary craniopharyngioma, of which ACP is most commonly observed in childhood (41). In ACP, the CTNNB1 mutation is the only consistent genomic alteration identified, which occurs in as many as 95% of cases and determines aberrant nuclear expression of β-catenin and canonical expression of WNT pathway target genes (42-44). ACP may develop at any point along the pituitary-hypothalamic region, from the intrasellar region to the third ventricle of the brain, but more than 50% originates at the level of the third ventricle floor, within the infundibulum and/or tuber cinereum regions (including the vital hypothalamus), and predominantly expand to the third ventricle cavity. Ectopic localization of cCP is rare (45-47). Treatment for cCP is given by neurosurgery with partial or total tumor resection, with or without radiotherapy, or in rare cases treatment of purely cystic lesions with interferon (48-50) or tocilizumab (51-53). Due to the well-described risk of damage to the hypothalamus with increasing extent of neurosurgical resection, the aim of neurosurgical intervention has been shifted to performing a limited resection followed by radiation therapy (54-57).

The overall survival of cCP is very good (58); however, the tumor or its treatment may induce considerable adverse effects, such as visual impairment and hypothalamic-pituitary damage. As a consequence of visual impairment (59, 60), morbid hypothalamic obesity (61-63), panhypopituitarism, second malignancies (64) cognitive impairment (33), neurobehavioral abnormalities (8), and/or vascular complications (65-67), life expectancy is decreased.

### Suprasellar Low-Grade Glioma

Pediatric LGG is one of the most common childhood central nervous system tumors, representing approximately 30% of all pediatric brain tumors (68). Although LGG are in general considered indolent tumors, they may give rise to serious functional disorders and some may have a more aggressive biology. When LGG is not surgically resectable or has more aggressive behavior, systemic treatment is needed. Often pediatric LGG requires several lines of treatment aiming to stabilize disease. When the LGG is located suprasellarly, hypothalamic-pituitary dysfunction may be present. Hypothalamic pituitary dysfunction in children with LGG may have different consequences dependent on age. Young children with suprasellar LGG may present with diencephalic syndrome (69, 70) and elevated concentrations of IGF-1, while older children and adolescents may develop hypothalamic hyperphagia with obesity and pituitary insufficiency (36).

### Other Suprasellar Tumors

Next to cCP and suprasellar LGG, other tumors in this region such as germ cell tumors (GCTs) may cause hypothalamic dysfunction (19, 71). GCTs, both germinoma and nongerminoma, may present with signs of hypothalamic-pituitary dysfunction due to the suprasellar tumor location. Due to involvement of or pressure to the pituitary stalk, AVP deficiency is often a presenting symptom. In boys with human chorionic gonadotropin–secreting nongerminoma, precocious puberty can be present, masking the simultaneous presence of a GH deficiency with inadequately normal growth velocity with ongoing development of sexual characteristics and advancing bone age. Treatment consists of surgery, chemotherapy, and radiation therapy, often resulting in panhypopituitarism with/without hypothalamic dysfunction (72). In a recent evaluation of the Dutch GCT cohort (n = 28), 75% were observed to have AVP deficiency and 57% to have anterior pituitary insufficiency at diagnosis but this increased to 100% after treatment. In 18% (5/26) of the GCT cohort, hypothalamic syndrome was diagnosed (31). Other tumors that may involve the suprasellar or hypothalamic area are pituitary adenoma (73, 74), prolactinoma (75), chordoma, and hypothalamic hamartoma (76-79). Nonneoplastic disease that may lead to hypothalamic dysfunction are Rathke cleft cysts (80).

The degree of hypothalamic dysfunction depends on the degree of hypothalamic involvement of the tumor as well as on the treatment given. It has been well described that neurosurgery increases the risk for hypothalamic dysfunction while the effect of radiation therapy on the function of the hypothalamus has been less well clarified (81). Laser ablation is a promising new technique to ablate epileptic foci in patients with hypothalamic hamartoma (gelastic seizures). During follow-up, disturbed sodium metabolism due to AVP deficiency or SIADH, hypothyroidism, weight gain, precocious puberty, hypocortisolism, and hyperphagia has been reported (78); however, the prevalence and severity of hypothalamic dysfunction in these cohorts have not been investigated thoroughly.

### Rapid Onset Obesity With Hypothalamic Dysfunction, Hypoventilation, and Autonomic Dysregulation Syndrome

One of the very rare causes of noncongenital hypothalamic dysfunction, of which currently around 200 cases have been reported globally, is ROHHAD syndrome (82). Children with ROHHAD typically present with rapid onset of (hypothalamic) obesity during the first years of life, followed by pituitary dysfunction, autonomic dysregulation, and respiratory distress due to central hypoventilation (83). In around 50% of patients with ROHHAD syndrome, a neuroendocrine tumor is found. Reported neuroendocrine tumors in ROHHAD syndrome are (ganglio)neuroblastoma or ganglioneuroma. The etiology of hypothalamic dysfunction in ROHHAD syndrome remains unclear, and no genetic or anatomical cause has been found. Current hypotheses include the hypothalamic dysfunction to be of autoimmune origin, due to the fact that antipituitary and antihypothalamus antibodies were identified in a patient with ROHHAD (84) and, more recently, the presence of anti-Zinc finger and SCAN domain containing 1 (ZSCAN1) antibodies were documented in patients with ROHHAD (85).

### Septo-Optic Dysplasia

SOD is a rare developmental disorder of the forebrain, optic pathway, and pituitary gland, with an incidence of about 1 in 10 000 births (86). It is defined by the presence of at least 2 of 3 components of the triad of optic nerve hypoplasia, hypopituitarism, or midline forebrain defects (eg, agenesis of the corpus callosum, absent septum pellucidum) (86, 87). The etiology of SOD as a congenital malformation disorder is multifactorial, with both genetic and environmental factors being implicated. For instance, the incidence of SOD is correlated with indices of deprivation such as unemployment and teenage pregnancies (86). Variants in a wide range of hypothalamo-pituitary developmental transcription factors have also been described in association with SOD. In around 1% to 4% of SOD cases a genetic cause can be identified such as HEX1 or GLI2 (88-90), but in the majority the underlying etiology is not identifiable (87, 91-95). Hypothalamic obesity develops in 31% of patients with SOD and can even occur in 12% of patients with isolated optic nerve hypoplasia (90). Associated neurological features are frequent (57% with bilateral optic nerve hypoplasia), ranging from focal neuro-ophthalmological deficits to developmental delay (96-98). Repetitive or restrictive behavioral difficulties and impairments of social communication can occur in a third of patients with SOD (99).

### Traumatic Brain Injury

TBI, which carries a considerable burden of disabilities, leads to a variety of endocrine dysfunctions in 28% to 69% of adult acute head-injured patients (37). In the acute posttraumatic phase, electrolyte disorders and adrenal insufficiency are critical conditions. Neurosurgical patients, particularly those prone to neurological damage, require prompt diagnosis. Hypopituitarism may be diagnosed months or years after a TBI event. Since GH and gonadotropin secretion are most frequently compromised, careful follow-up of growth and pubertal development is mandatory in children hospitalized for TBI.

### Hypophysitis and Hypothalamitis

Autoimmune hypophysitis or lymphocytic hypophysitis is a well-known autoimmunity-related pituitary disease. Autoimmune hypophysitis may occur as an isolated disease involving only the pituitary gland or it may be associated with other endocrine organ involvement such as autoimmune thyroid diseases (100-103). According to the anatomical involvement of the pituitary gland, autoimmune hypophysitis is traditionally described as lymphocytic adenohypophysitis, when only the anterior pituitary gland is involved, lymphocytic infundibulo-neurohypophysitis when the pituitary stalk and posterior pituitary gland are involved, and lymphocytic panhypophysitis, in which the entire pituitary gland including the anterior and posterior pituitary gland, and the pituitary stalk are involved (101, 103, 104). Patients with autoimmune hypophysitis may present with distinct radiological findings associated with variable degrees of hypopituitarism and/or hyperprolactinemia and AVP deficiency (103, 105). Until recently, hypothalamitis has not been recognized as a distinct entity and hypothalamic involvement presenting as a suprasellar mass has been interpreted as an upward continuation of autoimmune hypophysitis. In contrast, recent data suggest that autoimmune involvement of the hypothalamus presenting with a suprasellar mass may not be a component of autoimmune hypophysitis but an isolated autoimmune disease called hypothalamitis (106, 107).

## Management of Acquired Hypothalamic Syndrome

**Figure 5.**
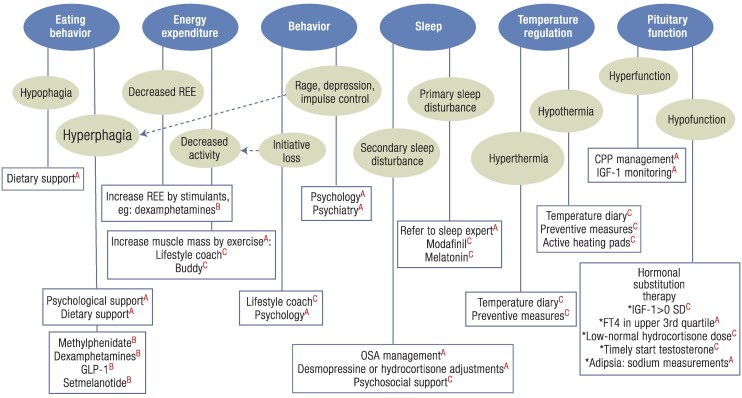
Algorithm for the management of (acquired) hypothalamic dysfunction. Hypothalamic dysfunction may lead to different signs and symptoms that may all contribute to the development of hypothalamic overweight or obesity and feelings of fatigue. By evaluating all 6 different clinical domains of hypothalamic dysfunction, with a step-wise approach, a personalized approach is enabled improving feelings of fatigue and decreasing obesity. Suggested interventions are categorized as existing guidelines ^A^, clinical trial or case series data ^B^, and expert opinion^C^. CPP, central precocious puberty; OSA, obstructive sleep apnea; REE, resting energy expenditure.

In this section, the management of acquired hypothalamic dysfunction is reviewed and an updated treatment algorithm is suggested (Fig. 5). In this updated treatment algorithm, when compared to the algorithm presented in 2019 (3), the focus is shifted from obesity management to hypothalamic dysfunction management. By focusing not only on BMI, but also on eating behavior, energy expenditure, sleep, temperature dysregulation, pituitary dysfunction, and overall behavior, the different underlying causes for the signs and symptoms of hypothalamic dysfunction will be addressed, which will not only contribute to improvement of BMI but also contribute to improved feelings of daily energy. With increased daily energy, daily activity and motivation for healthy lifestyle may also increase, with subsequent further improvement of BMI, improved participation in daily life (school or work), and decreased feelings of depression or gloominess. It is important to realize that hypothalamic dysfunction not only leads to obesity, but may also lead to nonalcoholic fatty liver disease (108), chronic fatigue (4), increased daytime sleepiness (5, 20, 109, 110), headaches (6), inactivity (7), and mood disorders (8), all of which may contribute to the development of obesity (9). As had been suggested by van Iersel et al (3), through a systemic approach to the different clinical consequences of hypothalamic dysfunction in different domains, the underlying cause for obesity may be better understood and doors for successful management can be opened. This applies not only to obesity management, but also to management of the other consequences of hypothalamic dysfunction, such as (chronic) fatigue. Hypothalamic dysfunction is, comparable to hypothalamic obesity, not one disease but requires personalized management. The new algorithm presented here aligns with the domains of the 2023 published diagnostic criteria (30), including the most recent insights and evidence for management of hypothalamic dysfunction in an integrated approach (see Fig. 5). It is important to acknowledge that the evidence for long-term outcomes of treatment for hypothalamic dysfunction remains limited. In future studies, this new algorithm may be implemented in registry-based follow-up or cohort studies to assess its long-term effectiveness. One of the most important barriers that must be overcome is often a low number of patients included in studies as a consequence of the rareness of the disease, and thus lack of power. By collaborating across countries and combining cohorts, such barriers may be overcome.

Because hypothalamic dysfunction is a rare disease, all children with hypothalamic dysfunction should be referred to an expert multidisciplinary team where the most up-to-date, interdisciplinary treatment can be offered (39, 80, 111). Van Schaik et al (112) recently showed that treatment with an experienced team may improve outcome. After centralization of care, a drop in occurrence of hypothalamic obesity in the Dutch cohort was observed from 33% to 12%.

### Management Requires a Multidisciplinary Approach and a Multidisciplinary Treatment Algorithm

The management of signs and clinical symptoms caused by (acquired) hypothalamic syndrome is complex, and due to its low prevalence, the evidence for different management strategies is low. For this reason, pediatric patients with hypothalamic dysfunction and/or hypothalamic syndrome should be referred to an expert center that has a multidisciplinary team (113). The signs and symptoms of (acquired) hypothalamic dysfunction can be, comparable to the diagnostic criteria (30), categorized in 6 different domains (see Fig. 5). By systematically evaluating each domain, the individual clinical characteristics of hypothalamic dysfunction will become clear, which will provide perspectives on novel options for personalized management. To enhance the utility of the proposed algorithm for the management of (acquired) hypothalamic dysfunction, suggested interventions have been categorized as based on existing guidelines, clinical trial or case series data, or expert opinion.

### Management of Eating Behavior and Treatment of Obesity

Hyperphagia in children with hypothalamic obesity can be very severe, with patients stealing food necessitating parents to lock their cupboards; however, hyperphagia is not present in all patients with hypothalamic obesity (30, 31). To endure the 24/7 hyperphagia, psychological counseling should be offered in addition to dietary and physical exercise counseling. With regard to diet, it is important to be aware that hypothalamic dysfunction is in general not a transient phenomenon. To have a lifelong diet may thus not be very encouraging; rather, speaking about a hypothalamic lifestyle may be more appealing. In a recent review on the feasibility, safety, and efficacy of dietary or lifestyle interventions for hypothalamic obesity, 12 studies were included reporting on 118 patients diagnosed with CP, ROHHAD syndrome, monogenic obesity, or PWS. Of these 12 studies, 4 reported that dietary interventions are feasible; however, difficulties were reported. Of the 7 studies that reported on efficacy of the diet, both “well-balanced restrictive caloric diets” (30% fat, 45% carbohydrates, and 25% protein) and various hypocaloric diets (8-10 kcal/cm/day) were considered effective with regard to weight stabilization or decrease (114).

Lifestyle interventions that have been reported so far have resulted in only a short-term, temporary BMI decrease, implying that additional coaching will be necessary for long-term effects. An important requisite for optimal lifestyle management is a strong home environment, especially due to the combination of 24/7 hyperphagia, pituitary dysfunction, and behavioral problems (115). In a recent evaluation of the German craniopharyngioma cohort (n = 291), BMI SD score (SDS) at last follow-up was correlated both to maternal BMI at diagnosis (*r* = 0.28; 95% CI, 0.17-0.38; *P* < .001) and paternal BMI at diagnosis (*r* = 0.3; 95% CI, 0.19-0.41; *P* < .001) (116). This implies that for proper obesity management, not only the patient but the whole family and caregivers (117) should be involved.

Several pharmaceutical interventions have been reported to be effective for hypothalamic hyperphagia, although this is also not one size fits all with, in each study, responders and nonresponders (3, 19, 118). In a recent small, adult cohort study including 26 patients with hypothalamic obesity, all but one lost weight with semaglutide treatment. Mean weight loss was 13.4 kg (loss of BMI of 4.4) after 12 months of treatment (119). A recent systematic review on glucagon-like peptide (GLP) receptor-1 treatment for hypothalamic obesity, in which 10 studies were included, reported that treatment with GLP-1 analogues may be effective and safe for weight control in hypothalamic obesity (120). In 2 small case series, dexamphetamines were reported to reduce BMI, increase REE, and improve energy level in children with hypothalamic obesity (121, 122). Targeted hormone replacement therapy with the melanocortin-4 receptor agonist, setmelanotide, has been shown to be very effective in children with monogenetic obesity such as Bardet-Biedl syndrome (123). Promising results were also found in a phase 2 trial in children with acquired obesity (124). Recently, the first results have been published online of a prospective placebo-controlled clinical trial including 120 patients with acquired hypothalamic obesity. This trial found that treatment with setmelanotide reduced mean BMI by −16.5% from baseline (n = 81) compared with a +3.3% BMI change for patients on placebo (n = 39) after 52 weeks (*P* < .0001). In addition, 80% of patients on setmelanotide achieved a BMI reduction of 5% or greater at 52 weeks (125).

Hyperinsulinemia can be present due to reduced vagal tone. This is an important aspect of hypothalamic obesity management, as this may result in increased storage of fat. In case of the presence of hyperglycemia, type 2 diabetes mellitus, or acanthosis nigricans, next to exercise, the first choice for pharmaceutical intervention is metformin (126); other options include diazoxide and metformin (127), Tesomet (128), octreotide (129-131), combination of oral phentermine and topiramate (Ph/T) (132, 133), or GLP-1 analogues (3, 17, 134-136).

Several central stimulants have been used off label for the treatment of hypothalamic obesity such as methylphenidate, phentermine, dextroamphetamine, mazindol, caffeine, and ephedrine (121, 122, 137-139), but data on these drugs are based on small studies with mixed results. Other medications such as sibutramine, a norepinephrine and serotonin inhibitor, lorcaserin, a serotonin 2C receptor agonist, and beloranib, a methionine aminopeptidase 2 inhibitor, were discontinued due to significant adverse events of thromboembolism and other safety concerns (140). The combination of bupropion, a norepinephrine-dopamine reuptake inhibitor, and naltrexone, an opioid receptor antagonist, has a black box warning of increased suicidal risk and ideation in young adults (141). There are no studies of bupropion-naltrexone in patients with hypothalamic obesity.

Ph/T might be another promising option, and it is US Food and Drug Administration approved for people aged 12 years or older with obesity (128, 129). However, this drug has not yet been tested in patients with hypothalamic obesity. Ph/T is a sympathomimetic amine combined with a GABAergic drug used to treat epilepsy. Children and adolescents with hypothalamic obesity have low sympathetic tone (142, 143), and thus may benefit from the stimulant-induced decrease in appetite (138, 144). Individuals with hypothalamic obesity can also have excess daytime sleepiness (5, 109, 145), which may contribute to impaired regulation of eating behavior and decreased physical activity (7) and may be targetable with stimulants. Reports of other types of stimulant use in individuals with hypothalamic obesity such as amphetamines have generally demonstrated weight loss or attenuation of weight gain (121, 122, 143, 146).

Tesofensine is a centrally acting triple-monoamine reuptake inhibitor that tackles low sympathetic tone in patients with hypothalamic obesity. Tesofensine results in reduction of caloric intake and weight loss by inhibiting the presynaptic reuptake of dopamine, serotonin, and noradrenaline, and inhibiting the dopamine active transporter (147). It is combined with the β-blocker metoprolol to reduce potential adverse effects due to adrenergic stimulation. Results from rodent studies suggest that indirect a-adrenergic and D1-dopaminergic stimulation contribute to reduction of food intake and body weight (148). A recent randomized, double-blind, placebo-controlled phase 2 trial including 21 randomized patients with tesomet treatment resulted in significant reductions in body weight, waist circumference, and glucose levels compared to placebo (128). The study results also showed that tesomet was safe and well tolerated and drug-related adverse events were mostly mild sleep disturbances, dry mouth, and headache.

**Figure 6.**
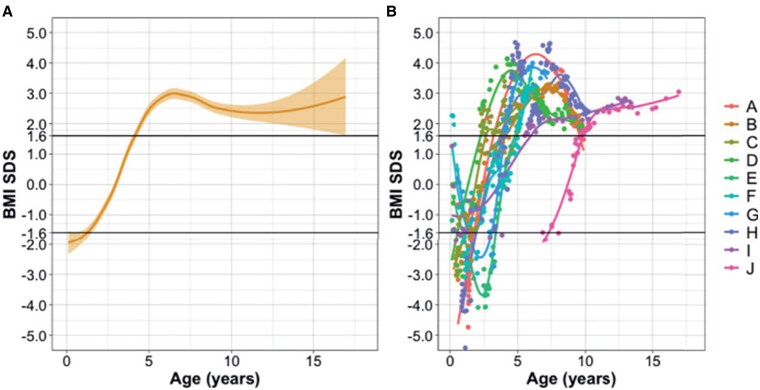
Underweight with hypothalamic hypophagia changing into overweight with hypothalamic hyperphagia in patients with low-grade glioma. Longitudinal body mass index (BMI) SDS over time. A, Logistic regression (LOESS) plot of all patients. x-axis: age in years at moment of BMI follow-up, y-axis: BMI SD score (SDS) corrected for age and sex. The beam shows 95% CI. B, Individualized BMI SDS data for each patient. This figure shows that the regression curve represents most of the patients, with only 2 outliers observed. Diencephalic syndrome defined as BMI SDS less than −1.6 SD. Overweight defined as BMI SDS greater than +1.6 SD. Reproduced from van Roessel et al, 2022 (36).

Hypophagia may also be encountered in patients with hypothalamic dysfunction and seems to be associated with young age in children with PWS and LGG (Fig. 6). In children with PWS, hypotonia and feeding problems may be present during the neonatal period, and in infants with suprasellar LGG diencephalic syndrome may be present. In children with CP, anorexia or hypophagia is rare, but has been described especially at younger age (149). In the German cohort, 11 of 485 patients with CP were observed to present with initial anorexia (150). In infants with PWS, eating behavior has been linked to the acylation of ghrelin. In young infants with PWS, acylated ghrelin (AG) concentrations have been found to be normal; however, unacylated ghrelin (UAG) levels were found to be increased. In older children with PWS, AG concentrations are increased when compared to normal obese children, and AG/AUG ratios are increased when compared to lean children. One of the causes of hypophagia may thus be higher levels of UAG, and a change in acylation may thus explain the change in time (151). Management of hypophagia is mainly dietary. Because hypophagia may change into hyperphagia, education and psychological support is mandatory for patients and parents to notice any timely shifts in eating behavior.

Bariatric interventions demonstrated notable effectiveness in weight loss and safety in patients with CP (152-154). In 2013, Bretault et al (153) reviewed in their individual-level meta-analysis a total of 21 cases of CP and found significant weight loss at 6 months and 12 months after bariatric surgery (−0.9%TWL/6 months, −15.1%TWL/12 months). The greatest weight reduction was achieved after Roux-en-Y gastric bypass (RYGB). Compared to results in normal controls, total weight reduction during 2 years was lower for other interventions but RYGB (153). In pediatric patients, the application of nonreversible bariatric techniques such as bypass interventions is ethically and legally controversial and should be made by multidisciplinary teams in the context of national/international trials (154). The Endocrine Society's Clinical Practice Guideline for Treating Pediatric Obesity (155) suggests that only adolescents with morbid obesity and advanced pubertal development, who have reached near final or final height and are adherent to diet and exercise interventions, should be considered for bariatric surgery. It is also important to be aware of the postoperative complications after bariatric surgery, including diarrhea, dumping syndrome, and iron-deficiency anemia following RYGB, folic acid and vitamin D deficiency, impaired effectiveness of oral desmopressin medication following sleeve gastrectomy, and dysphagia and vomiting following laparoscopic gastric banding, all of which may have more profound effects on patients with hypothalamic obesity (154). As patients with hypothalamic obesity have varying hypothalamic and pituitary functions, the net benefit from bariatric interventions on hypothalamic obesity is uncertain, and they should be evaluated individually to decide whether to operate and by which bariatric procedure.

Deep brain stimulation is a novel technique with potential benefit in the treatment of hypothalamic obesity after CP (156). Deep brain stimulation causes a “functional” lesion or modulation of a brain network by high-frequency stimulation. Promising experiences have been reported for deep brain stimulation in patients with Parkinson disease, epilepsy, dystonia, and obsessive-compulsive disorders. First experiences in deep brain stimulation treatment of eating disorders such as anorexia nervosa and obesity were promising. In 3 studies, deep brain stimulation was used for treatment of hypothalamic obesity in 6 patients (5 patients with PWS and 1 patient with CP). Targets of deep brain stimulation included the nucleus accumbens and the lateral hypothalamic area. In the reported CP patient, nucleus accumbens deep brain stimulation led to a decrease in BMI (−8.7%) (157). No severe side effects were observed in these trials. Reports on long-term follow-up are still missing.

Truncal vagotomy alone or in combination with bariatric interventions may be effective in hypothalamic obesity, by reducing the efferent output both to β cells and adipocytes (158). Lustig (158) has performed laparoscopic truncal vagotomy in 4 individuals with hypothalamic obesity, with early results being supportive of this procedure, and with relatively few complications or side effects. However, reports on long-term effects and tolerability of truncal vagotomy are missing.

### Decreased Energy Expenditure and Its Management

Decreased energy expenditure can be caused by a decreased REE as a consequence of distorted hypothalamic efferent signals or due to decreased physical activity resulting in decreased muscle mass (122). REE can be easily measured by ventilated hood calorimetry (16). To assess REE may help not only in understanding changes in BMI but also guide dietary advice and personalized management. Patients with hypothalamic dysfunction frequently also experience initiative loss, for which an exercise coach or buddy may help. By increasing muscle mass (testosterone, long-term training) and increasing body temperature (with active heating in case of temperature < 35 °C), REE can be increased. To optimize energy expenditure, all pituitary deficiencies should be supplemented, including GH and timely thyroxine (T4). In addition, to increase REE, pharmacological treatment with dexamphetamines can be considered (121, 122).

### Management of Hypothalamic-Associated Behavioral Problems.

Children with hypothalamic dysfunction need guidance from specialized outpatient clinics including psychologists and psychiatrists. More studies are needed on understanding, better prediction of, and improving neurocognitive and social behavior (8). As oxytocin may be lowered or deficient, a pilot randomized trial was conducted to investigate its effect on behavior. Some benefits of oxytocin for anxiety and impulsivity were observed, but it did not improve obesity in individuals with hypothalamic obesity (159). A randomized, placebo-controlled study of 8 weeks’ intranasal oxytocin for children and young adults with tumor-induced hypothalamic obesity (NCT 02849743) is ongoing. While safety data so far are reassuring, neuropsychological effects of intranasal oxytocin on psychosocial behavior and emotion regulation have been observed in CP patients (160-162). Further clinical trials are warranted to analyze and understand the optimal drug formulation and dosing regimen.

### Management of Hypothalamic Sleep Disorders

For detection of sleep problems a good history is often sufficient, which can be objectified by actigraphy. If a sleep problem is suspected, it is recommended to further analyze the problem using the diagnostic algorithm of hypothalamic sleep problems as suggested by van Schaik et al (25). This includes obstructive sleep apnea syndrome diagnostics, actigraphy, and history-taking for other clues for sleep disorders including polyuria or nocturia as a sign of badly regulated AVP deficiency. Müller et al (29) analyzed sleep disorders in patients with CP and hypothalamic obesity and observed a high rate of patients presenting with polysomnographic signs of secondary narcolepsy. Medical treatment by central stimulating agents improved sleep disorders in CP patients with secondary narcolepsy.

### Management of Hypothalamic Temperature Dysregulation

Hypothalamic dysfunction may cause severe hypothermia or hyperthermia without signs of infection (19). Current management is preventive and practical. For children with hypothermia, active heating pads can provide sufficient warmth. For children with temperature spikes and periods of hyperthermia, no treatment is available except for dressing lightly. When hypothermia or hyperthermia is less than 35.5 C or greater than 38 C, hydrocortisone stress dosages should be considered. Management of temperature dysregulation is mainly focused on preventive measures such as dressing warm when cold outside or staying in the house in front of the air conditioning during hot summers. Prevention of temperature changes may result in an increase of daily energy and a reduction of headaches.

### Management of Pituitary Dysfunction in Patients With Hypothalamic Syndrome

Most children with hypothalamic dysfunction experience pituitary insufficiency (19). Young children may however also present with central precocious puberty or increased IGF-1 concentrations. In case of central precocious puberty, treatment is indicated. The elevated IGF-1 concentrations normalize in time in most cases (21).

In case of hypopituitarism, adequate supplementation is mandatory. Lack of GH, thyroid hormone, cortisol, or gonadal hormones may result in chronic fatigue, headaches, reduced activity, and increase of BMI. GH substitution therapy is needed in GH-deficient patients to restore normal body composition and increase bone mineral density. For hypothalamic patients specifically, GH treatment has been shown to decrease BMI and improve body composition, especially when given during childhood (163, 164). International consensus has stated GH treatment is safe in children with GH deficiency after a benign tumor such as pituitary adenoma or CP, and there is no need to delay initiation of GH treatment. For high-grade tumor types, it is advisable to wait at least 1 year following the end of tumor treatment and only when radiologically confirmed stability is achieved, considering that tumor relapse is highest during the first 12 months after cancer treatment (165).

Central hypothyroidism can be missed when free T4 serum concentrations are in the lower half of the reference range (166-168). In case the free T4 concentration drops by more than 20% over time, central hypothyroidism must be suspected, necessitating treatment with levothyroxine (169). Levothyroxine should be substituted at a dose sufficient to achieve free T4 serum levels in the mid to upper half of the reference range (170).

In the case of frequently encountered tumor- and/or treatment-associated adrenocorticotropin deficiency in CP patients, hydrocortisone-substitution therapy and flexible-dose adaptation with regard to stress situations are mandatory (168, 171). Cortisol deficiency may lead to fatigue, headaches, and vomiting, and untreated hypocortisolism can even be life-threatening in the context of an acute adrenal crisis. It has been shown that in children with acquired hypothalamic dysfunction, 11β-hydroxysteroid dehydrogenase type 1 activity is increased (172), which is why in some cases, lower hydrocortisone doses may be considered in children with acquired hypothalamic dysfunction than for children without hypothalamic disease (6-8 mg/m^2^). Nonphysiological overreplacement of hydrocortisone may promote the development of obesity. A well-balanced and adapted dosage of hydrocortisone is beneficial for stabilizing BMI and functional capacity as well as preventing risks of adrenal crises and hypoglycemia. Patient and parental training on the perception of stress situations and appropriate and rapid intervention in life-threatening emergency situations such as acute adrenal crisis is of vital importance (173).

Male hypogonadism, resulting in low serum testosterone concentrations, has been associated with an adverse metabolic phenotype and even increased mortality (174-176). Timely and adequate testosterone substitution therapy will help to improve body composition with increased muscle mass and decreased free fat mass. Increased muscle mass will subsequently increase basal metabolic rate and help exercise possibilities, which will in turn increase bone density, reduce body fat and cardiac risk factors, and promote cognitive function and psychological well-being. Importantly, not all patients with hypothalamic dysfunction have pituitary dysfunction (30).

## Conclusions

Acquired hypothalamic dysfunction and hypothalamic syndrome are rare but can be devastating. For optimal management, children require a multidisciplinary approach in an expert center. Management should focus not only on obesity or weight reduction, but also on fatigue, behavior, sleep, temperature regulation, and optimal treatment of pituitary dysfunction. The uncontrollable hyperphagia makes it not only an individual problem, but in many cases, a dramatic family affair. Hypothalamic syndrome is not one disease but requires individualized treatment. Characterization of hypothalamic dysfunction per individual in the 6 domains will not only improve diagnostics but also enables the development of an individual treatment plan. New exciting treatments are upcoming for hyperphagia and hypothalamic obesity, so a promising future may lie ahead for our patients. Future research may focus on the domain of temperature regulation disorders and adipsic AVP deficiency due to the fact that these consequences of hypothalamic dysfunction can control daily life, requiring continuous monitoring of the patient, with no treatment available yet. Because hypothalamic syndrome is a rare disease, international collaborations with joint studies is the way forward.

## Abbreviations

ACP: adamantinomatous craniopharyngioma
AG: acylated ghrelin
AGRP: agouti-related peptide
AVP: arginine vasopressin
BMI: body mass index
cCP: childhood-onset craniopharyngioma
GCT: germ cell tumor
GH: growth hormone
GLP: glucagon-like peptide
IGF-1: insulin-like growth factor-1
LGG: low-grade glioma
MCG: monoaminergic cell group
mREE: measured resting energy expenditure
NPY: neuropeptide Y
Ph/T: combination of oral phentermine and topiramate
POMC: pro-opiomelanocortin
pREE: predicted resting energy expenditure
PWS: Prader-Will syndrome
ROHHAD: rapid-onset obesity with hypothalamic dysregulation, hypoventilation, and autonomic dysregulation
RYGB: Roux-en-Y gastric bypass
SIADH: syndrome of inappropriate AVP secretion
SOD: septo-optic dysplasia
T4: thyroxine
TBI: traumatic brain injury
UAG: unacylated ghrelin
VLPO: ventrolateral preoptic nucleus

## References

[1] Kreier F, Swaab DF. History of hypothalamic research: “the spring of primitive existence”. Handb Clin Neurol. 2021;179:7‐43. 34225985 10.1016/B978-0-12-819975-6.00031-5

[2] Placzek M, Chinnaiya K, Kim DW, Blackshaw S. Control of tuberal hypothalamic development and its implications in metabolic disorders. Nat Rev Endocrinol. 2024;21:118‐130. 39313573 10.1038/s41574-024-01036-1 PMC11864813

[3] Van Iersel L, Brokke KE, Adan RAH, Bulthuis LCM, Van Den Akker ELT, Van Santen HM. Pathophysiology and individualized treatment of hypothalamic obesity following craniopharyngioma and other suprasellar tumors: a systematic review. Endocr Rev. 2018;40(1):193‐235. 10.1210/er.2018-00017 30247642

[4] Beckhaus J, Özyurt J, Mehren A, Friedrich C, Müller HL. Fatigue in patients with hypothalamic syndrome—a cross-sectional analysis of the German childhood-onset craniopharyngioma cohort. EJC Paediatr Oncol. 2024;4:100174.

[5] Müller HL . Increased daytime sleepiness in patients with childhood craniopharyngioma and hypothalamic tumor involvement: review of the literature and perspectives. Int J Endocrinol. 2010;2010:1‐7. 10.1155/2010/519607 PMC3017941 21234339

[6] Hoffmann A, Boekhoff S, Gebhardt U, et al History before diagnosis in childhood craniopharyngioma: associations with initial presentation and long-term prognosis. Eur J Endocrinol. 2015;173(6):853‐862. 26392473 10.1530/EJE-15-0709

[7] Harz KJ, Müller HL, Waldeck E, Pudel V, Roth C. Obesity in patients with craniopharyngioma: assessment of food intake and movement counts indicating physical activity. J Clin Endocrinol Metab. 2003;88(11):5227‐5231. 14602754 10.1210/jc.2002-021797

[8] Özyurt J, Thiel CM, Lorenzen A, et al Neuropsychological outcome in patients with childhood craniopharyngioma and hypothalamic involvement. J Pediatr. 2014;164(4):876‐881.e4. 24507865 10.1016/j.jpeds.2013.12.010

[9] van Roessel IMAA, de Graaf JP, Biermasz NR, Charmandari E, van Santen HM. Acquired hypothalamic dysfunction in childhood: “what do patients need?”—an endo-ERN survey. Endocr Connect. 2023;12:e230147. 37531603 10.1530/EC-23-0147 PMC10503223

[10] Fountas A, Coulden A, Fernández-García S, Tsermoulas G, Allotey J, Karavitaki N. Central diabetes insipidus (vasopressin deficiency) after surgery for pituitary tumours: a systematic review and meta-analysis. Eur J Endocrinol. 2024;191(1):S1‐S13. 38996052 10.1093/ejendo/lvae084

[11] Tran LT, Park S, Kim SK, Lee JS, Kim KW, Kwon O. Hypothalamic control of energy expenditure and thermogenesis. Exp Mol Med. 2022;54(4):358‐369. 35301430 10.1038/s12276-022-00741-z PMC9076616

[12] Heymsfield SB, Avena NM, Baier L, et al Hyperphagia: current concepts and future directions proceedings of the 2nd international conference on hyperphagia. Obesity. 2014;22(S1):S1‐S17. 10.1002/oby.20646 PMC4159941 24574081

[13] Pop MG, Crivii CB, Opincariu I. Introduction to the hypothalamus: correlates from animal studies. In: *The Human Hypothalamus.* 2021:3-6.

[14] Brown CH, Bains JS, Ludwig M, Stern JE. Physiological regulation of magnocellular neurosecretory cell activity: integration of intrinsic, local and afferent mechanisms. J Neuroendocrinol. 2013;25(8):678‐710. 23701531 10.1111/jne.12051 PMC3852704

[15] Gan HW, Cerbone M, Dattani MT. Appetite- and weight-regulating neuroendocrine circuitry in hypothalamic obesity. Endocr Rev. 2024;45(3):309‐342. 38019584 10.1210/endrev/bnad033 PMC11074800

[16] Van Schaik J, Burghard M, Lequin MH, et al Resting energy expenditure in children at risk of hypothalamic dysfunction. Endocr Connect. 2022;11(8):e220276. 35904233 10.1530/EC-22-0276 PMC9346331

[17] Jílková A, Lampová B, Kádě O, et al Resting energy expenditure in patients with extreme obesity: comparison of the harris-benedict equation with indirect calorimetry. J Clin Med. 2024 Oct 8;13(19):5993. 39408053 10.3390/jcm13195993 PMC11478319

[18] Van Roessel IMAA, Van Schaik J, Kleinlugtenbelt LB, et al Physical activity, health-related fitness, and physical performance in children with acquired hypothalamic dysfunction. Support Care Cancer. 2025;33(4):295. 40100427 10.1007/s00520-025-09361-5 PMC11920002

[19] Müller HL, Tauber M, Lawson EA, et al Hypothalamic syndrome. Nat Rev Dis Primers. 2022;8(1):24. 35449162 10.1038/s41572-022-00351-z

[20] Arima H, Cheetham T, Christ-Crain M, et al Changing the name of diabetes insipidus: a position statement of the working group for renaming diabetes insipidus. J Clin Endocrinol Metab. 2022;108(1):1‐3. 36355385 10.1210/clinem/dgac547 PMC9759163

[21] van Schaik J, van Roessel IMAA, Bos ID, et al Elevated IGF-1 concentrations in children with low grade glioma: a descriptive analysis in a retrospective national cohort. J Neuroendocrinol. 2023;35:e13317. 37439273 10.1111/jne.13317

[22] Müller HL, Handwerker G, Wollny B, Faldum A, Sörensen N. Melatonin secretion and increased daytime sleepiness in childhood craniopharyngioma patients. J Clin Endocrinol Metab. 2002;87(8):3993‐3996. 12161549 10.1210/jcem.87.8.8751

[23] Gapstur R, Gross CR, Ness K. Factors associated with sleep-wake disturbances in child and adult survivors of pediatric brain tumors: a review. Oncol Nurs Forum. 2009;36(6):723‐731. 19887361 10.1188/09.ONF.723-731

[24] Foschi M, Sambati L, Zoli M, et al Site and type of craniopharyngiomas impact differently on 24-hour circadian rhythms and surgical outcome. A neurophysiological evaluation. Auton Neurosci. 2017;208:126‐130. 28843459 10.1016/j.autneu.2017.08.006

[25] van Schaik J, Pillen S, van Litsenburg RRL, et al The importance of specialized sleep investigations in children with a suprasellar tumor. Pituitary. 2020;23:613‐621. 32691357 10.1007/s11102-020-01065-9 PMC7585563

[26] Marcus CL, Trescher WH, Halbower AC, Lutz J. Secondary narcolepsy in children with brain tumors. Sleep. 2002;25(4):435‐439. 12071545

[27] Ono D, Yamanaka A. Hypothalamic regulation of the sleep/wake cycle. Neurosci Res. 2017;118:74‐81. 28526553 10.1016/j.neures.2017.03.013

[28] Lu J, Greco MA, Shiromani P, Saper CB. Effect of lesions of the ventrolateral preoptic nucleus on NREM and REM sleep. J Neurosci. 2000;20(10):3830‐3842. 10804223 10.1523/JNEUROSCI.20-10-03830.2000 PMC6772663

[29] Müller HL, Müller-Stöver S, Gebhardt U, Kolb R, Sörensen N, Handwerker G. Secondary narcolepsy may be a causative factor of increased daytime sleepiness in obese childhood craniopharyngioma patients. J Pediatr Endocrinol Metab. 2006;19(Suppl 1):423‐429. 16700320 10.1055/s-2006-974095

[30] van Santen HM, van Schaik J, van Roessel IMAA, Beckhaus J, Boekhoff S, Müller HL. Diagnostic criteria for the hypothalamic syndrome in childhood. Eur J Endocrinol. 2023;188(2):214‐225. 10.1093/ejendo/lvad009 36737045

[31] van Roessel IMAA, Hulsmann SC, Schouten-van Meeteren AYN, et al The many different clinical faces of acquired hypothalamic dysfunction: a retrospective cohort study in the Netherlands. EClinicalMedicine. 2025;85:103313. Doi: 10.1016/j.eclinm.2025.103313 PMC12270709 40686687

[32] Mehren A, Özyurt J, Zu Klampen P, Boekhoff S, Thiel CM, Müller HL. Self- and informant-rated apathy in patients with childhood-onset craniopharyngioma. J Neurooncol. 2018;140(1):27‐35. 29971569 10.1007/s11060-018-2936-z

[33] Özyurt J, Müller HL, Thiel CM. A systematic review of cognitive performance in patients with childhood craniopharyngioma. J Neurooncol. 2015;125(1):9‐21. 26369768 10.1007/s11060-015-1885-z

[34] Dattani MT, Martinez-Barbera J-P, Thomas PQ, et al Molecular genetics of septo-optic dysplasia. Horm Res Paediatr. 2000;53(Suppl. 1):26‐33. 10.1159/000053201 10895039

[35] Tauber M, Hoybye C. Endocrine disorders in prader-willi syndrome: a model to understand and treat hypothalamic dysfunction. Lancet Diabetes Endocrinol. 2021;9(4):235‐246. 33647242 10.1016/S2213-8587(21)00002-4

[36] van Roessel IMAA, Schouten-van Meeteren AYN, Meijer L, Hoving EW, Bakker B, van Santen HM. Transition from diencephalic syndrome to hypothalamic obesity in children with suprasellar low grade glioma: a case series. Front Endocrinol (Lausanne). 2022;13:846124. 35464054 10.3389/fendo.2022.846124 PMC9019925

[37] Einaudi S, Bondone C. The effects of head trauma on hypothalamic–pituitary function in children and adolescents. Curr Opin Pediatr. 2007;19(4):465‐470. 17630613 10.1097/MOP.0b013e3281ab6eeb

[38] Lee JM, Shin J, Kim S, et al Rapid-onset obesity with hypoventilation, hypothalamic, autonomic dysregulation, and neuroendocrine tumors (ROHHADNET) syndrome: a systematic review. Biomed Res Int. 2018;2018:1‐17. 10.1155/2018/1250721 PMC6280256 30584530

[39] van Schaik J, Verrico A, Müller HL, et al Craniopharyngioma standard clinical practice recommendations. 2021 CRCTU-PRT-QCD-001; 2021

[40] Witte J, Surmann B, Batram M, et al Hypothalamic obesity: epidemiology in rare sellar/suprasellar tumors—a German claims database analysis. J Neuroendocrinol. 2024;36(12):e13439. 39191454 10.1111/jne.13439 PMC11646665

[41] Santagata S, Kleinschmidt DeMasters B, Müller HL, Nishioka H, Takashi T, Yamada S. Adamantinamatous craniopharyngioma. In WHO Classification of Tumours Editorial Board, ed. Central Nervous System Tumours [Internet]. WHO classification of tumours series, 5th ed. vol. 6. International Agency for Research on Cancer; 2021.

[42] Martinez-Barbera JP . Molecular and cellular pathogenesis of adamantinomatous craniopharyngioma. Neuropathol Appl Neurobiol. 2015;41(6):721‐732. 25611703 10.1111/nan.12226 PMC4949713

[43] Martinez-Barbera JP, Buslei R. Adamantinomatous craniopharyngioma: pathology, molecular genetics and mouse models. J Pediatr Endocrinol Metab. 2015;28(1-2):7‐17. 25503464 10.1515/jpem-2014-0442

[44] Martinez-Barbera JP, Andoniadou CL. Biological behaviour of craniopharyngiomas. Neuroendocrinology. 2020;110(9-10):797‐804. 32126562 10.1159/000506904

[45] Hoffmann A, Brentrup A, Müller HL. First report on spinal metastasis in childhood-onset craniopharyngioma. J Neurooncol. 2016;129(1):193‐194. 27278607 10.1007/s11060-016-2160-7

[46] Nogueira J, Sobreiro Silva J, Marques R, Antunes C, Pereira R, Afonso Filipe M. Ectopic craniopharyngioma recurrence: a case report and literature review. Cureus. 2024;16:e69607. 39429347 10.7759/cureus.69607 PMC11486633

[47] Kordes U, Flitsch J, Hagel C, et al Ectopic craniopharyngioma. Klin Padiatr. 2011;223(3):176‐177. 21462099 10.1055/s-0031-1273743

[48] Hedrich C, Patel P, Haider L, et al Feasibility, tolerability, and first experience of intracystic treatment with peginterferon alfa-2a in patients with cystic craniopharyngioma. Front Oncol. 2024;14:1401761. 39050573 10.3389/fonc.2024.1401761 PMC11266088

[49] Cavalheiro S, Di Rocco C, Valenzuela S, et al Craniopharyngiomas: intratumoral chemotherapy with interferon-α: a multicenter preliminary study with 60 cases. Neurosurg Focus. 2010;28(4):E12. 10.3171/2010.1.FOCUS09310 20367356

[50] Kilday J-P, Caldarelli M, Massimi L, et al Intracystic interferon-alpha in pediatric craniopharyngioma patients: an international multicenter assessment on behalf of SIOPE and ISPN. Neuro Oncol. 2017;19(10):1398‐1407. 28499018 10.1093/neuonc/nox056 PMC5596165

[51] Webb LM, Okuno SH, Ransom RC, et al Recurrent adamantinomatous craniopharyngioma stabilized with tocilizumab and bevacizumab: illustrative case. J Neurosurg Case Lessons. 2025;9(2):CASE24410. 39805108 10.3171/CASE24410 PMC11734617

[52] de Vos-Kerkhof E, Buis DR, Lequin MH, et al Tocilizumab for the fifth progression of cystic childhood craniopharyngioma—a case report. Front Endocrinol (Lausanne). 2023;14:1225734. 37886643 10.3389/fendo.2023.1225734 PMC10598752

[53] Grob S, Mirsky DM, Donson AM, et al Targeting IL-6 is a potential treatment for primary cystic craniopharyngioma. Front Oncol. 2019;9:791. 31497533 10.3389/fonc.2019.00791 PMC6712354

[54] Bogusz A, Boekhoff S, Warmuth-Metz M, Calaminus G, Eveslage M, Müller HL. Posterior hypothalamus-sparing surgery improves outcome after childhood craniopharyngioma. Endocr Connect. 2019;8(5):481‐492. 30925462 10.1530/EC-19-0074 PMC6479199

[55] Müller HL . More or less? Treatment strategies in childhood craniopharyngioma. Child's Nervous System. 2006;22(2):156‐157. 10.1007/s00381-005-1192-7 16320024

[56] Hoffmann A, Warmth-Metz M, Gebhardt U, et al Childhood craniopharyngioma—changes of treatment strategies in the trials KRANIOPHARYNGEOM 2000/2007. Klin Padiatr. 2014;226(3):161‐168. 24819386 10.1055/s-0034-1368785

[57] Elowe-Gruau E, Beltrand J, Brauner R, et al Childhood craniopharyngioma: hypothalamus-sparing surgery decreases the risk of obesity. J Clin Endocrinol Metab. 2013;98(6):2376‐2382. 23633208 10.1210/jc.2012-3928

[58] Sterkenburg AS, Hoffmann A, Gebhardt U, Warmuth-Metz M, Daubenbüchel AMM, Müller HL. Survival, hypothalamic obesity, and neuropsychological/psychosocial status after childhood-onset craniopharyngioma: newly reported long-term outcomes. Neuro Oncol. 2015;17(7):1029‐1038. 25838139 10.1093/neuonc/nov044 PMC5654354

[59] Nuijts MA, Veldhuis N, Stegeman I, et al Visual functions in children with craniopharyngioma at diagnosis: a systematic review. PLoS One. 2020;15(10):e0240016. 33002047 10.1371/journal.pone.0240016 PMC7529266

[60] Sowithayasakul P, Beckhaus J, Boekhoff S, Friedrich C, Calaminus G, Müller HL. Vision-related quality of life in patients with childhood-onset craniopharyngioma. Sci Rep. 2023;13(1):19599. 37949931 10.1038/s41598-023-46532-y PMC10638396

[61] Beckhaus J, Friedrich C, Boekhoff S, et al Outcome after pediatric craniopharyngioma: the role of age at diagnosis and hypothalamic damage. Eur J Endocrinol. 2023;188(3):300‐309. 10.1093/ejendo/lvad027 36857103

[62] Müller HL, Emser A, Faldum A, et al Longitudinal study on growth and body mass Index before and after diagnosis of childhood craniopharyngioma. J Clin Endocrinol Metab. 2004;89(7):3298‐3305. 15240606 10.1210/jc.2003-031751

[63] Müller HL, Bueb K, Bartels U, et al Obesity after childhood craniopharyngioma—german multicenter study on pre-operative risk factors and quality of life. Klin Padiatr. 2001;213(4):244‐249. 11528558 10.1055/s-2001-16855

[64] Newhauser WD, Durante M. Assessing the risk of second malignancies after modern radiotherapy. Nat Rev Cancer. 2011;11(6):438‐448. 21593785 10.1038/nrc3069 PMC4101897

[65] Beckhaus J, Friedrich C, Müller HL. Vascular morbidity and mortality in craniopharyngioma patients—a scoping review. Cancers (Basel). 2024;16(6):1099. 38539434 10.3390/cancers16061099 PMC10969212

[66] Boekhoff S, Bison B, Genzel D, et al Cerebral infarction in childhood-onset craniopharyngioma patients: results of KRANIOPHARYNGEOM 2007. Front Oncol. 2021;11:698150. 34336685 10.3389/fonc.2021.698150 PMC8317984

[67] Lucas JT, Faught AM, Hsu CY, et al Pre- and posttherapy risk factors for vasculopathy in pediatric patients with craniopharyngioma treated with surgery and proton radiation therapy. Int J Radiation Oncol Biol Phys. 2022;113(1):152‐160. 10.1016/j.ijrobp.2021.12.172 PMC9018579 34990778

[68] Ryall S, Tabori U, Hawkins C. Pediatric low-grade glioma in the era of molecular diagnostics. Acta Neuropathol Commun. 2020;8(1):30. 32164789 10.1186/s40478-020-00902-z PMC7066826

[69] Kilday J-P, Bartels U, Huang A, et al Favorable survival and metabolic outcome for children with diencephalic syndrome using a radiation-sparing approach. J Neurooncol. 2014;116(1):195‐204. 24218181 10.1007/s11060-013-1284-2

[70] Gnekow AK, Falkenstein F, von Hornstein S, et al Long-term follow-up of the multicenter, multidisciplinary treatment study HIT-LGG-1996 for low-grade glioma in children and adolescents of the German speaking society of pediatric oncology and hematology. Neuro Oncol. 2012;14(10):1265‐1284. 22942186 10.1093/neuonc/nos202 PMC3452343

[71] Gan H-W, Bulwer C, Spoudeas H. Pituitary and hypothalamic tumor syndromes in childhood. In: Feingold KR, Anawalt B, Boyce A, Chrousos G, de Herder WW, Dhatariya K, Dungan K, Grossman A, Hershman JM, Hofland J, Kalra S, Kaltsas G, Koch C, Kopp P, Korbonits M, Kovacs CS, Kuohung W, Laferrère B, McGee EA, McLachlan R, Morley JE, New M, Purnell J, Sahay R, Singer F, Stratakis CA, Trence DL, Wilson DP, eds. Endotext [Internet]. MDText.com, Inc; 2000. 25905376

[72] van Iersel L, van Santen HM, Potter B, et al Clinical impact of hypothalamic-pituitary disorders after conformal radiation therapy for pediatric low-grade glioma or ependymoma. Pediatr Blood Cancer. 2020;67:e28723. 33037871 10.1002/pbc.28723

[73] Molitch ME . Diagnosis and treatment of pituitary adenomas. JAMA. 2017;317(5):516. 28170483 10.1001/jama.2016.19699

[74] Fernandez A, Karavitaki N, Wass JAH. Prevalence of pituitary adenomas: a community-based, cross-sectional study in banbury (oxfordshire, UK). Clin Endocrinol (Oxf). 2010;72(3):377‐382. 19650784 10.1111/j.1365-2265.2009.03667.x

[75] Hoffmann A, Adelmann S, Lohle K, Claviez A, Müller HL. Pediatric prolactinoma: initial presentation, treatment, and long-term prognosis. Eur J Pediatr. 2018;177(1):125‐132. 29168011 10.1007/s00431-017-3042-5

[76] Rizzi M, Consales A, Tramacere I, et al Surgical and radiosurgical treatment of hypothalamic hamartoma: the Italian experience between 2011 and 2021. Epilepsia Open. 2024;9(4):1493‐1501. 38926936 10.1002/epi4.12989 PMC11296090

[77] Hinojosa J, Candela-Cantó S, Becerra V, et al Multimodal approach for the treatment of Complex hypothalamic hamartomas. Adv Tech Stand Neurosurg. 2024;50:119‐145. 38592529 10.1007/978-3-031-53578-9_4

[78] Ahmed S, Nadeem ZA, Kamran U, et al Magnetic resonance-guided Laser interstitial thermal therapy in the management of hypothalamic hamartomas: a systematic review and meta-analysis. World Neurosurg. 2024;190:463‐469.e6. 39122113 10.1016/j.wneu.2024.08.009

[79] Shirozu H, Masuda H, Kameyama S. A proposed new classification system of hypothalamic hamartomas in the era of stereotactic ablation surgery. J Neurosurg. 2024;142(4):956‐967. 39671576 10.3171/2024.7.JNS24560

[80] Müller HL, Gebhardt U, Faldum A, et al Xanthogranuloma, Rathke's cyst, and childhood craniopharyngioma: results of prospective multinational studies of children and adolescents with rare sellar malformations. J Clin Endocrinol Metab. 2012;97(11):3935‐3943. 22969141 10.1210/jc.2012-2069

[81] Paulissen JMJ, Zegers CML, Houben RM, et al Radiotherapy-induced hypothalamic-pituitary axis dysfunction in adult brain, head and neck and skull base tumor patients—a systematic review and meta-analysis. Clin Transl Radiat Oncol. 2025;51:100900. 39801827 10.1016/j.ctro.2024.100900 PMC11721507

[82] Khaytin I, Victor AK, Barclay SF, et al Rapid-onset obesity with hypothalamic dysfunction, hypoventilation, and autonomic dysregulation (ROHHAD): a collaborative review of the current understanding. Clin Auton Res. 2023;33(3):251‐268. 37162653 10.1007/s10286-023-00936-y

[83] Harvengt J, Gernay C, Mastouri M, et al ROHHAD(NET) syndrome: systematic review of the clinical timeline and recommendations for diagnosis and prognosis. J Clin Endocrinol Metab. 2020;105(7):2119‐2131. 10.1210/clinem/dgaa247 32407531

[84] Giacomozzi C, Guaraldi F, Cambiaso P, et al Anti-hypothalamus and anti-pituitary auto­antibodies in ROHHAD syndrome: additional evidence supporting an autoimmune etiopathogenesis. Horm Res Paediatr. 2019;92(2):124‐132. 31039576 10.1159/000499163

[85] Serafim AB, Olivé-Cirera G, Ortega-González Á, et al Antibodies against ZSCAN1 in pediatric and adult patients with non-paraneoplastic ROHHAD syndrome. Neurol Neuroimmunol Neuroinflamm. 2024;11(5):e200276. 38917381 10.1212/NXI.0000000000200276 PMC11204383

[86] Patel L, McNally RJQ, Harrison E, Lloyd IC, Clayton PE. Geographical distribution of optic nerve hypoplasia and septo-optic dysplasia in northwest England. J Pediatr. 2006;148(1):85‐88. 16423603 10.1016/j.jpeds.2005.07.031

[87] Webb EA, Dattani MT. Septo-optic dysplasia. Eur J Hum Genet. 2010;18(4):393‐397. 19623216 10.1038/ejhg.2009.125 PMC2987262

[88] McNay DE, Turton JP, Kelberman D, et al HESX1 mutations are an uncommon cause of septooptic dysplasia and hypopituitarism. J Clin Endocrinol Metab. 2007;92(2):691‐697. 17148560 10.1210/jc.2006-1609

[89] Paulo SS, Fernandes-Rosa FL, Turatti W, et al Sonic hedgehog mutations are not a common cause of congenital hypopituitarism in the absence of complex midline cerebral defects. Clin Endocrinol (Oxf). 2015;82(4):562‐569. 25056824 10.1111/cen.12565

[90] Cerbone M, Güemes M, Wade A, Improda N, Dattani M. Endocrine morbidity in midline brain defects: differences between septo-optic dysplasia and related disorders. EClinicalMedicine. 2020;19:100224. 32140665 10.1016/j.eclinm.2019.11.017 PMC7046495

[91] McCabe MJ, Hu Y, Gregory LC, et al Novel application of luciferase assay for the in vitro functional assessment of KAL1 variants in three females with septo-optic dysplasia (SOD). Mol Cell Endocrinol. 2015;417:63‐72. 26375424 10.1016/j.mce.2015.09.010 PMC4646839

[92] McCabe MJ, Gaston-Massuet C, Tziaferi V, et al Novel *FGF8* mutations associated with recessive holoprosencephaly, craniofacial defects, and hypothalamo-pituitary dysfunction. J Clin Endocrinol Metab. 2011;96(10):E1709‐E1718. 21832120 10.1210/jc.2011-0454 PMC3417283

[93] McCabe MJ, Gaston-Massuet C, Gregory LC, et al Variations in *PROKR2*, but not *PROK2*, are associated with hypopituitarism and septo-optic dysplasia. J Clin Endocrinol Metab. 2013;98(3):E547‐E557. 23386640 10.1210/jc.2012-3067 PMC3612801

[94] Gaston-Massuet C, McCabe MJ, Scagliotti V, et al Transcription factor 7-like 1 is involved in hypothalamo–pituitary axis development in mice and humans. Proc Natl Acad Sci U S A. 2016;113(5):E548‐E557. 26764381 10.1073/pnas.1503346113 PMC4747739

[95] Dattani MT, Martinez-Barbera J-P, Thomas PQ, et al Mutations in the homeobox gene HESX1/hesx1 associated with septo-optic dysplasia in human and mouse. Nat Genet. 1998;19(2):125‐133. 9620767 10.1038/477

[96] Salman MS, Ruth CA, Yogendran MS, Lix LM. Morbidities and comorbidities associated with optic nerve hypoplasia and septo-optic-pituitary dysplasia. Dev Med Child Neurol. 2025;67:941‐952. 39804979 10.1111/dmcn.16235

[97] Haddad NG, Eugster EA. Hypopituitarism and neurodevelopmental abnormalities in relation to central nervous system structural defects in children with optic nerve hypoplasia. J Pediatr Endocrinol Metab. 2005;18(9):853‐858. 16279362 10.1515/jpem.2005.18.9.853

[98] Lourdes Garcia M, Ty EB, Taban M, Rothner AD, Rogers D, Traboulsi EI. Systemic and ocular findings in 100 patients with optic nerve hypoplasia. J Child Neurol. 2006;21(11):949‐956. 17092460 10.1177/08830738060210111701

[99] Parr JR, Dale NJ, Shaffer LM, Salt AT. Social communication difficulties and autism spectrum disorder in young children with optic nerve hypoplasia and/or septo-optic dysplasia. Dev Med Child Neurol. 2010;52(10):917‐921. 20370811 10.1111/j.1469-8749.2010.03664.x

[100] Gubbi S, Hannah-Shmouni F, Verbalis JG, Koch CA. Hypophysitis: an update on the novel forms, diagnosis and management of disorders of pituitary inflammation. Best Pract Res Clin Endocrinol Metab. 2019;33(6):101371. 31866206 10.1016/j.beem.2019.101371 PMC7078033

[101] Gubbi S, Hannah-Shmouni F, Stratakis CA, Koch CA. Primary hypophysitis and other autoimmune disorders of the sellar and suprasellar regions. Rev Endocr Metab Disord. 2018;19(4):335‐347. 30547288 10.1007/s11154-018-9480-1

[102] Bayram F, Keleştimur F, Öztürk F, Selçuklu A, Patiroğlu TE, Beyhan Z. Lymphocytic hypophysitis in a patient with Graves’ disease. J Endocrinol Invest. 1998;21(3):193‐197. 9591217 10.1007/BF03347301

[103] Bellastella A, Bizzarro A, Coronella C, Bellastella G, Sinisi A, De Bellis A. Lymphocytic hypophysitis: a rare or underestimated disease? Eur J Endocrinol. 2003;149(5):363‐376. 14585081 10.1530/eje.0.1490363

[104] Caturegli P, Lupi I, Landek-Salgado M, Kimura H, Rose NR. Pituitary autoimmunity: 30 years later. Autoimmun Rev. 2008;7(8):631‐637. 18774118 10.1016/j.autrev.2008.04.016 PMC3383826

[105] Unlühizarci K, Bayram F, Colak R, et al Distinct radiological and clinical appearance of lymphocytic hypophysitis. J Clin Endocrinol Metab. 2001;86(5):1861‐1864. 11344171 10.1210/jcem.86.5.7440

[106] Tshuma N, Glynn N, Evanson J, Powles T, Drake WM. Hypothalamitis and severe hypothalamic dysfunction associated with anti–programmed cell death ligand 1 antibody treatment. Eur J Cancer. 2018;104:247‐249. 30377030 10.1016/j.ejca.2018.09.016

[107] Bertulli L, Bertani GA, Gianelli U, Mantovani G, Rampini PM, Locatelli M. Long-standing isolated autoimmune hypothalamitis diagnosed with endoscopic transventricular biopsy. World Neurosurg. 2017;105:1036.e5‐1036.e9. 10.1016/j.wneu.2017.06.055 28625907

[108] Hoffmann A, Bootsveld K, Gebhardt U, Daubenbüchel AMM, Sterkenburg AS, Müller HL. Nonalcoholic fatty liver disease and fatigue in long-term survivors of childhood-onset craniopharyngioma. Eur J Endocrinol. 2015;173(3):389‐397. 26088821 10.1530/EJE-15-0422

[109] Müller HL, Handwerker G, Gebhardt U, et al Melatonin treatment in obese patients with childhood craniopharyngioma and increased daytime sleepiness. Cancer Causes Control. 2006;17(4):583‐589. 16596314 10.1007/s10552-005-9012-7

[110] Gebhardt U, Handwerker G, Müller-Stöver S, Sörensen N, Müller H. Secondary narcolepsy may be a causative factor of increased daytime sleepiness in obese patients with childhood craniopharyngioma. Neuropediatrics. 2006;37(06):423‐429. 10.1055/s-2006-974095 16700320

[111] Müller HL, Gebhardt U, Teske C, et al Post-operative hypothalamic lesions and obesity in childhood craniopharyngioma: results of the multinational prospective trial KRANIOPHARYNGEOM 2000 after 3-year follow-up. Eur J Endocrinol. 2011;165(1):17‐24. 21490122 10.1530/EJE-11-0158

[112] Van Schaik J, Schouten-van Meeteren AYN, Vos-Kerkhof E, et al Treatment and outcome of the Dutch childhood craniopharyngioma cohort study; first results after centralization of care. Neuro Oncol. 2023;25:2250‐2261. 37381692 10.1093/neuonc/noad112 PMC10708930

[113] van Schaik J, Hoving EW, Müller HL, van Santen HM. Hypothalamic-pituitary outcome after treatment for childhood craniopharyngioma. Front Horm Res. 2021;54:47‐57. 33965963 10.1159/000515318

[114] Van Roessel IMAA, Van Den Brink M, Dekker J, Ruitenburg-van Essen BG, Tissing WJE, van Santen HM. Feasibility, safety, and efficacy of dietary or lifestyle interventions for hypothalamic obesity: a systematic review. Clinical Nutrition. 2024;43(8):1798‐1811. 38955055 10.1016/j.clnu.2024.05.028

[115] Meijneke RWH, Schouten-Van Meeteren AYN, De Boer NY, et al Hypothalamic obesity after treatment for craniopharyngioma: the importance of the home environment. J Pediatr Endocrinol Metab. 2015;28(1-2):1‐2. 25381948 10.1515/jpem-2014-0338

[116] Beckhaus J, Eveslage M, Bison B, Friedrich C, Müller HL. Impact of parental body mass index at diagnosis on obesity in survivors of pediatric craniopharyngioma. Endocr Connect. 2024;13(8):e240126. 38904467 10.1530/EC-24-0126 PMC11301543

[117] Kayadjanian N, Hsu EA, Wood AM, Carson DS. Caregiver burden and its relationship to health-related quality of life in craniopharyngioma survivors. J Clin Endocrinol Metab. 2023;109(1):e76‐e87. 37597173 10.1210/clinem/dgad488 PMC10735386

[118] Dimitri P . Treatment of acquired hypothalamic obesity: now and the future. Front Endocrinol (Lausanne). 2022;13:846880. 35464063 10.3389/fendo.2022.846880 PMC9019363

[119] Svendstrup M, Rasmussen AK, Kistorp C, Klose M, Andreassen M. Semaglutide treatment of hypothalamic obesity—a real-life data study. Pituitary. 2024:27;685‐692. 39120810 10.1007/s11102-024-01429-5 PMC11513754

[120] Ng VWW, Gerard G, Koh JJK, Loke KY, Lee YS, Ng NBH. The role of glucagon-like peptide 1 receptor agonists for weight control in individuals with acquired hypothalamic obesity—a systematic review. Clin Obes. 2024;14(3):e12642. 38273176 10.1111/cob.12642

[121] Denzer C, Denzer F, Lennerz BS, Vollbach H, Lustig RH, Wabitsch M. Treatment of hypothalamic obesity with dextroamphetamine: a case series. Obes Facts. 2019;12(1):91‐102. 30844799 10.1159/000495851 PMC6465734

[122] van Schaik J, Welling MS, de Groot CJ, et al Dextroamphetamine treatment in children with hypothalamic obesity. Front Endocrinol (Lausanne). 2022;13:845937. 35355559 10.3389/fendo.2022.845937 PMC8959487

[123] Trapp CM, Censani M. Setmelanotide: a promising advancement for pediatric patients with rare forms of genetic obesity. Curr Opin Endocrinol Diabetes Obes. 2023;30(2):136‐140. 36722447 10.1097/MED.0000000000000798 PMC9973437

[124] Roth CL, Scimia C, Shoemaker AH, et al Setmelanotide for the treatment of acquired hypothalamic obesity: a phase 2, open-label, multicentre trial. Lancet Diabetes Endocrinol. 2024;12(6):380‐389. 38697184 10.1016/S2213-8587(24)00087-1

[125] https://ir.rhythmtx.com/news-releases/news-release-details/rhythm-pharmaceuticals-announces-pivotal-phase-3-transcend-trial

[126] Kalina MA, Wilczek M, Kalina-Faska B, Skała-Zamorowska E, Mandera M, Małecka Tendera E. Carbohydrate-lipid profile and use of metformin with micronized fenofibrate in reducing metabolic consequences of craniopharyngioma treatment in children: single institution experience. J Pediatr Endocrinol Metab. 2014;28(1-2):1‐2. 10.1515/jpem-2014-0425 25536662

[127] Hamilton J, Conwell L, Syme C, Ahmet A, Jeffery A, Daneman D. Hypothalamic obesity following craniopharyngioma surgery: results of a pilot trial of combined diazoxide and metformin therapy. Int J Pediatr Endocrinol. 2011;2011(1):417949. 21603206 10.1155/2011/417949 PMC3198743

[128] Huynh K, Klose M, Krogsgaard K, et al Randomized controlled trial of tesomet for weight loss in hypothalamic obesity. Eur J Endocrinol. 2022;186(6):687‐700. 35294397 10.1530/EJE-21-0972 PMC9175551

[129] Lustig RH, Rose SR, Burghen GA, et al Hypothalamic obesity caused by cranial insult in children: altered glucose and insulin dynamics and reversal by a somatostatin agonist. J Pediatr. 1999;135(2):162‐168. 10431109 10.1016/s0022-3476(99)70017-x

[130] Lustig RH, Greenway F, Velasquez-Mieyer P, et al A multicenter, randomized, double-blind, placebo-controlled, dose-finding trial of a long-acting formulation of octreotide in promoting weight loss in obese adults with insulin hypersecretion. Int J Obes. 2006;30(2):331‐341. 10.1038/sj.ijo.0803074 PMC1540404 16158082

[131] Lustig RH, Hinds PS, Ringwald-Smith K, et al Octreotide therapy of pediatric hypothalamic obesity: a double-blind, placebo-controlled trial. J Clin Endocrinol Metab. 2003;88(6):2586‐2592. 12788859 10.1210/jc.2002-030003

[132] Hsia DS, Gosselin NH, Williams J, et al A randomized, double-blind, placebo-controlled, pharmacokinetic and pharmacodynamic study of a fixed-dose combination of phentermine/topiramate in adolescents with obesity. Diabetes Obes Metab. 2020;22(4):480‐491. 31696603 10.1111/dom.13910

[133] Allison DB, Gadde KM, Garvey WT, et al Controlled-release phentermine/topiramate in severely obese adults: a randomized controlled trial (EQUIP). Obesity. 2012;20(2):330‐342. 22051941 10.1038/oby.2011.330 PMC3270297

[134] Roth CL, Zenno A. Treatment of hypothalamic obesity in people with hypothalamic injury: new drugs are on the horizon. Front Endocrinol (Lausanne). 2023;14:1256514. 37780616 10.3389/fendo.2023.1256514 PMC10533996

[135] Roth CL, Perez FA, Whitlock KB, et al A phase 3 randomized clinical trial using a once-weekly glucagon-like peptide-1 receptor agonist in adolescents and young adults with hypothalamic obesity. Diabetes Obes Metab. 2021;23(2):363‐373. 33026160 10.1111/dom.14224 PMC7821019

[136] Gjersdal E, Larsen LB, Ettrup KS, et al Semaglutide as a promising treatment for hypothalamic obesity: a six-month case series on four females with craniopharyngioma. Pituitary. 2024;27(5):723‐730. 39088138 10.1007/s11102-024-01426-8 PMC11513775

[137] Greenway FL, Bray GA. Treatment of hypothalamic obesity with caffeine and ephedrine. Endocr Pract. 2008;14(6):697‐703. 18996788 10.4158/EP.14.6.697

[138] Elfers CT, Roth CL. Effects of methylphenidate on weight gain and food intake in hypothalamic obesity. Front Endocrinol (Lausanne). 2011;2:78. 22649386 10.3389/fendo.2011.00078 PMC3355874

[139] Sadatomo T, Sakoda K, Yamanaka M, Kutsuna M, Kurisu K. Mazindol administration improved hyperphagia after surgery for craniopharyngioma. Case report. Neurol Med Chir (Tokyo). 2001;41(4):210‐212. 11381681 10.2176/nmc.41.210

[140] Danielsson P, Janson A, Norgren S, Marcus C. Impact sibutramine therapy in children with hypothalamic obesity or obesity with aggravating syndromes. J Clin Endocrinol Metab. 2007;92(11):4101‐4106. 17726084 10.1210/jc.2007-0826

[141] Singhal V, Sella AC, Malhotra S. Pharmacotherapy in pediatric obesity: current evidence and landscape. Curr Opin Endocrinol Diabetes Obes. 2021;28(1):55‐63. 33186194 10.1097/MED.0000000000000587 PMC8082722

[142] Roth CL, Hunneman DH, Gebhardt U, Stoffel-Wagner B, Reinehr T, Müller HL. Reduced sympathetic metabolites in urine of obese patients with craniopharyngioma. Pediatr Res. 2007;61(4):496‐501. 17515878 10.1203/pdr.0b013e3180332cd6

[143] Son JW, Kim S. Comprehensive review of current and upcoming anti-obesity drugs. Diabetes Metab J. 2020;44(6):802‐818. 33389955 10.4093/dmj.2020.0258 PMC7801751

[144] Mason PW, Krawiecki N, Meacham LR. The use of dextroamphetamine to treat obesity and hyperphagia in children treated for craniopharyngioma. Arch Pediatr Adolesc Med. 2002;156(9):887. 12197795 10.1001/archpedi.156.9.887

[145] Mandrell BN, LaRosa K, Hancock D, et al Predictors of narcolepsy and hypersomnia due to medical disorder in pediatric craniopharyngioma. J Neurooncol. 2020;148(2):307‐316. 32346835 10.1007/s11060-020-03519-3

[146] Horne VE, Bielamowicz K, Nguyen J, et al Methylphenidate improves weight control in childhood brain tumor survivors with hypothalamic obesity. Pediatr Blood Cancer. 2020;67(7):e28379. 32383818 10.1002/pbc.28379

[147] Hansen HH, Hansen G, Tang-Christensen M, et al The novel triple monoamine reuptake inhibitor tesofensine induces sustained weight loss and improves glycemic control in the diet-induced obese rat: comparison to sibutramine and rimonabant. Eur J Pharmacol. 2010;636(1-3):88‐95. 20385125 10.1016/j.ejphar.2010.03.026

[148] Axel AMD, Mikkelsen JD, Hansen HH. Tesofensine, a novel triple monoamine reuptake inhibitor, induces appetite suppression by indirect stimulation of α1 adrenoceptor and dopamine D1 receptor pathways in the diet-induced obese rat. Neuropsychopharmacology. 2010;35(7):1464‐1476. 20200509 10.1038/npp.2010.16 PMC3055463

[149] Balcázar-Hernández L, Vargas-Ortega G, Valverde-García Y, Mendoza-Zubieta V, González-Virla B. Anorexia-cachexia syndrome-like hypothalamic neuroendocrine dysfunction in a patient with a papillary craniopharyngioma. Endocrinol Diabetes Metab Case Rep. 2017;2017:17-0018. 10.1530/EDM-17-0018 PMC5409936 28469924

[150] Hoffmann A, Gebhardt U, Sterkenburg AS, Warmuth-Metz M, Müller HL. Diencephalic syndrome in childhood craniopharyngioma—results of German multicenter studies on 485 long-term survivors of childhood craniopharyngioma. J Clin Endocrinol Metab. 2014;99(11):3972‐3977. 25077898 10.1210/jc.2014-1680

[151] Vlaardingerbroek H, van den Akker ELT, Hokken-Koelega ACS. Appetite- and weight-inducing and -inhibiting neuroendocrine factors in prader-willi syndrome, bardet-biedl syndrome and craniopharyngioma versus anorexia nervosa. Endocr Connect. 2021;10(5):R175‐R188. 33884958 10.1530/EC-21-0111 PMC8183618

[152] Müller H, Gebhardt U, Wessel V, et al First experiences with laparoscopic adjustable gastric banding (LAGB) in the treatment of patients with childhood craniopharyngioma and morbid obesity. Klin Padiatr. 2007;219(6):323‐325. 18050042 10.1055/s-2007-985848

[153] Bretault M, Boillot A, Muzard L, et al Bariatric surgery following treatment for craniopharyngioma: a systematic review and individual-level data meta-analysis. J Clin Endocrinol Metab. 2013;98(6):2239‐2246. 23533238 10.1210/jc.2012-4184

[154] Müller HL . Bariatric interventions in craniopharyngioma patients—best choice or last option for treatment of hypothalamic obesity? J Clin Endocrinol Metab. 2022;107(1):e426‐e428. 34331765 10.1210/clinem/dgab567

[155] Styne DM, Arslanian SA, Connor EL, et al Pediatric obesity—assessment, treatment, and prevention: an endocrine society clinical practice guideline. J Clin Endocrinol Metab. 2017;102(3):709‐757. 28359099 10.1210/jc.2016-2573 PMC6283429

[156] Dassen AR, van Schaik J, van den Munckhof P, Schuurman PR, Hoving EW, van Santen HM. Could deep brain stimulation be a possible solution for acquired hypothalamic obesity? Heliyon. 2023;9(3):e14411. 36967879 10.1016/j.heliyon.2023.e14411 PMC10036662

[157] Harat M, Rudaś M, Zieliński P, Birska J, Sokal P. Nucleus accumbens stimulation in pathological obesity. Neurol Neurochir Pol. 2016;50(3):207‐210. 27154450 10.1016/j.pjnns.2016.01.014

[158] Lustig RH . Hypothalamic obesity after craniopharyngioma: mechanisms, diagnosis, and treatment. Front Endocrinol (Lausanne). 2011;2:60. 22654817 10.3389/fendo.2011.00060 PMC3356006

[159] McCormack SE, Wang Z, Wade KL, et al A pilot randomized clinical trial of intranasal oxytocin to promote weight loss in individuals with hypothalamic obesity. J Endocr Soc. 2023;7(5):bvad037. 37153702 10.1210/jendso/bvad037 PMC10154909

[160] Daubenbüchel AM, Özyurt J, Boekhoff S, Warmuth-Metz M, Eveslage M, Müller HL. Eating behaviour and oxytocin in patients with childhood-onset craniopharyngioma and different grades of hypothalamic involvement. Pediatr Obes. 2019;14(9):e12527. 31013553 10.1111/ijpo.12527

[161] Daubenbüchel AMM, Hoffmann A, Eveslage M, et al Oxytocin in survivors of childhood-onset craniopharyngioma. Endocrine. 2016;54(2):524‐531. 27585663 10.1007/s12020-016-1084-5

[162] Hoffmann A, Özyurt J, Lohle K, Reichel J, Thiel CM, Müller HL. First experiences with neuropsychological effects of oxytocin administration in childhood-onset craniopharyngioma. Endocrine. 2017;56(1):175‐185. 28213803 10.1007/s12020-017-1257-x

[163] Heinks K, Boekhoff S, Hoffmann A, et al Quality of life and growth after childhood craniopharyngioma: results of the multinational trial KRANIOPHARYNGEOM 2007. Endocrine. 2018;59(2):364‐372. 29230635 10.1007/s12020-017-1489-9

[164] Boekhoff S, Bogusz A, Sterkenburg AS, Eveslage M, Müller HL. Long-term effects of growth hormone replacement therapy in childhood-onset craniopharyngioma: results of the German Craniopharyngioma Registry (HIT-Endo). Eur J Endocrinol. 2018;179(5):331‐341. 30139824 10.1530/EJE-18-0505

[165] Boguszewski MCS, Boguszewski CL, Chemaitilly W, et al Safety of growth hormone replacement in survivors of cancer and intracranial and pituitary tumours: a consensus statement. Eur J Endocrinol. 2022;186(6):P35‐P52. 35319491 10.1530/EJE-21-1186 PMC9066587

[166] Van Iersel L, Van Santen HM, Zandwijken GRJ, Zwaveling-Soonawala N, Hokken-Koelega ACS, Van Trotsenburg ASP. Low FT4 concentrations around the start of recombinant human growth hormone treatment: predictor of congenital structural hypothalamic-pituitary abnormalities? Horm Res Paediatr. 2018;89(2):98‐107. 29402813 10.1159/000486033

[167] van Iersel L, Clement SC, Schouten-Van Meeteren AYN, et al Declining free thyroxine levels over time in irradiated childhood brain tumor survivors. Endocr Connect. 2018;7(12):1322‐1332. 30400062 10.1530/EC-18-0311 PMC6280587

[168] van Iersel L, Mulder RL, Denzer C, et al Hypothalamic-pituitary and other endocrine surveillance among childhood cancer survivors. Endocr Rev. 2021;43:794‐823. 10.1210/endrev/bnab040 34962573

[169] Van Iersel L, Xu J, Potter BS, et al Clinical importance of free thyroxine concentration decline after radiotherapy for pediatric and adolescent brain tumors. J Clin Endocrinol Metab. 2019;104(11):4998‐5007. 31173083 10.1210/jc.2019-00539

[170] Fleseriu M, Hashim IA, Karavitaki N, et al Hormonal replacement in hypopituitarism in adults: an endocrine society clinical practice guideline. J Clin Endocrinol Metab. 2016;101(11):3888‐3921. 27736313 10.1210/jc.2016-2118

[171] Zhou Z, Zhang S, Hu F. Endocrine disorder in patients with craniopharyngioma. Front Neurol. 2021;12:737743. 34925209 10.3389/fneur.2021.737743 PMC8675636

[172] Tiosano D, Eisentein I, Militianu D, Chrousos GP, Hochberg Z. 11 beta-hydroxysteroid dehydrogenase activity in hypothalamic obesity. J Clin Endocrinol Metab. 2003;88(1):379‐384. 12519880 10.1210/jc.2002-020511

[173] Bereket A . Postoperative and long-term endocrinologic complications of craniopharyngioma. Horm Res Paediatr. 2020;93(9-10):497‐509. 33794526 10.1159/000515347

[174] Blackwell KM, Buckingham H, Paul KK, Uddin H, Jehle D von K, Blackwell TA. Benefits of testosterone replacement therapy in hypogonadal males. J Am Board Fam Med. 2024;37(5):816‐825. 39978846 10.3122/jabfm.2024.240025R1

[175] Jaiswal V, Sawhney A, Nebuwa C, et al Association between testosterone replacement therapy and cardiovascular outcomes: a meta-analysis of 30 randomized controlled trials. Prog Cardiovasc Dis. 20ik24;85:45‐53. 38589271 10.1016/j.pcad.2024.04.001

[176] Miller C, Madden-Doyle L, Jayasena C, McIlroy M, Sherlock M, O’Reilly MW. *Mechanisms in endocrinology:* hypogonadism and metabolic health in men—novel insights into pathophysiology. Eur J Endocrinol. 2024;191(6):R1‐R17. 10.1093/ejendo/lvae128 39344641

